# Cascade synthesis of 2,4-disulfonylpyrroles by the sulfonylation/[2 + 3]-cycloaddition reactions of *gem*-dibromoalkenes with arylsulfonyl methyl isocyanides†

**DOI:** 10.1039/d0ra10451e

**Published:** 2021-04-12

**Authors:** Parvin Salehi, Zahra Tanbakouchian, Noushin Farajinia-Lehi, Morteza Shiri

**Affiliations:** Department of Chemistry, Faculty of Physics and Chemistry, Alzahra University Vanak Tehran 1993893973 Iran mshiri@alzahra.ac.ir

## Abstract

An efficient cascade reaction involving sulfonylation and [2 + 3]-cycloaddition reactions of *gem*-dibromoalkenes with arylsulfonyl methyl isocyanides was described for the synthesis of 3-aryl-2,4-disulfonyl-1*H*-pyrroles. The main highlight of this study is the introduction of a new dual-functional reactivity of arylsulfonyl methyl isocyanides as the sulfonyl source as well as a 1,3-dipolar reagent in the same reaction. This system is facilitated by Cs_2_CO_3_ mediation in DMSO and 100 °C conditions.

## Abstract 2

An efficient cascade reaction involving sulfonylation and [2 + 3]-cycloaddition reactions of *gem*-dibromoalkenes with arylsulfonyl methyl isocyanides was described for the synthesis of 3-aryl-2,4-disulfonyl-1*H*-pyrroles.

## Graphical abstract

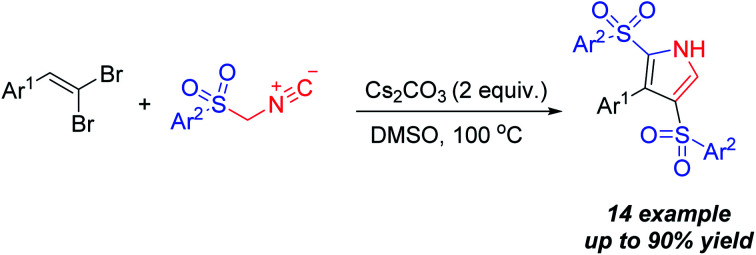

## Introduction

Since its first isolation from bone pyrolysis in 1857, the pyrrole unit was identified as a fundamental substructure of numerous important natural and bioactive unnatural products.^1^ Lamellarin R as an anti-cancer and anti-HIV alkaloid,^2^ and other active pyrrolizidine alkaloids, chlorophyll, cobalamin (vitamin B_12_), heme and bile pigments are some of the well-known naturally occurring pyrrole derivatives.^3^ Hence, numerous syntheses for substituted pyrroles have been reported including classical Hantzsch,^4^ Knorr^5^ and Paal–Knorr^6^ methods and some alternative procedures such as aza-Wittig, coupling, condensation and annulation reactions.^7^ Owing to the practicality and commercial accessibility of their compounds, isocyanides and π systems have been demonstrated to be attractive functionalities for heterocyclic cycloaddition reactions that due to appropriate atom-economy are considered as an ideal approach for the direct synthesis of substituted pyrroles.^8^

**Fig. 1:**
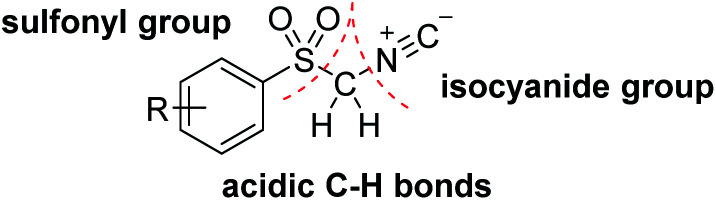
Structure of TosMIC.

In particular, recently, activated isocyanides, including α-acidic isocyanides with additional functionalities owing to their 1,3-dipolar nature and efficient cooperation in [3 + 2] cycloaddition reactions have attracted considerable attention of numerous synthetic researchers.^9^ Amongst these structures, arylsulfonyl methyl isocyanides (ASMIC), particularly *p*-toluenesulfonylmethyl isocyanide (TosMIC), are one of the most impressive reagents from both the stability and reactivity point of views.^10^ TosMIC, as an odorless derivative of isocyanides can afford a multitude of reactions *via* its three different functionalities of α-acidic carbon, isocyanide and sulfonyl groups.^11^ Two features of less sterically congested α-C–H bonds and enhanced electrophilicity as a consequence of additional sulfonyl functionality distinguish this reactive reagent from other alkyl isocyanides (Fig. 1).^12,13^

**Scheme 1:**
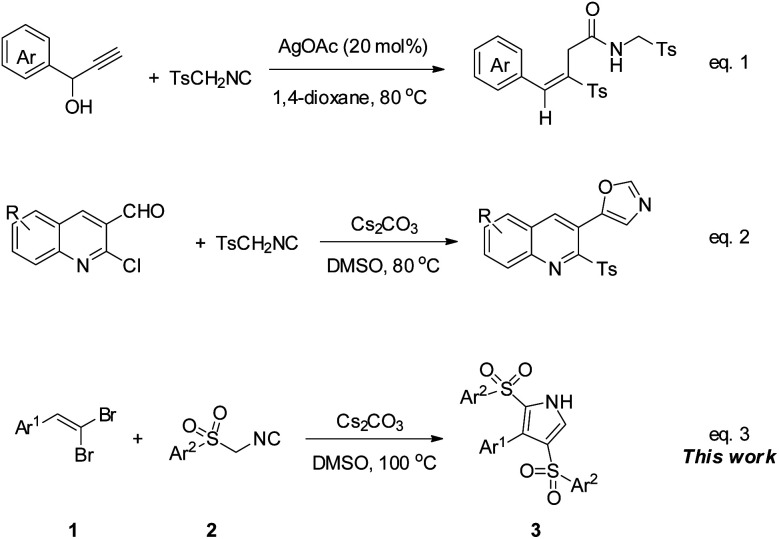
ASMIC as a dual-functional reactive starting material in the same reaction.

Moreover, sulfonyl function as a leaving group can offer TosMIC an efficient source of sulfonyl group, whereby it can act as a dual reagent and/or as a sulfonylation reagent.^12^ In this regard, Bi *et al.* introduced the dual role of TosMIC in a cascade reaction with propargylic alcohols, wherein TosMIC acted as a reagent for allenylative and sulfonylation in the presence of Ag_2_CO_3_ to give (*E*)-vinyl sulfones (Scheme 1, eqn (1)).^14^ Also, recently, our group investigated the behavior of TosMIC in treatment by 2-chloroquinoline-3-carbaldehydes, and the result was in tandem with the van Leusen/ring-closing procedure and tosylation to create 5-(2-tosylquinolin-3-yl)oxazoles, which testified to the dual-functional reactivity of mentioned isocyanide (Scheme 1, eqn (2)).^15^

As our ongoing effort to explore the novel synthetic application of 1,1-dibromoalkenes, we have previously investigated the reaction of this alkenes with two reagents of alkyl isocyanides and sodium sulfinates, which in the former, 3-(hetero)arylpropynamides furnished from a palladium-catalyzed cross-coupling reaction,^16^ and the latter involved a sulfonylation to yield the desired 1-bromo-1-sulfonylalkenes.^17^*gem*-Dibromo-1-alkenes, which can be easily generated from the treatment of aldehydes or ketones with CBr_4_/PPh_3_,^18^ can act as an alternative resource of activated alkene and alkyne moieties.^19^ Encouraged by these findings, we envisaged that arylsulfonyl methyl isocyanides might react with 1,1-dibromoalkenes as a dual functional reagent, both as a [1,3]-dipolar and as a sulfonyl source in a cascade process. To our delight, 3-aryl-2,4-disulfonyl-1*H*-pyrroles were isolated up to 90% yield from the mentioned reaction under Cs_2_CO_3_ condition at 100 °C (Scheme 1, eqn (3)).

## Results and discussion

**Scheme 2:**
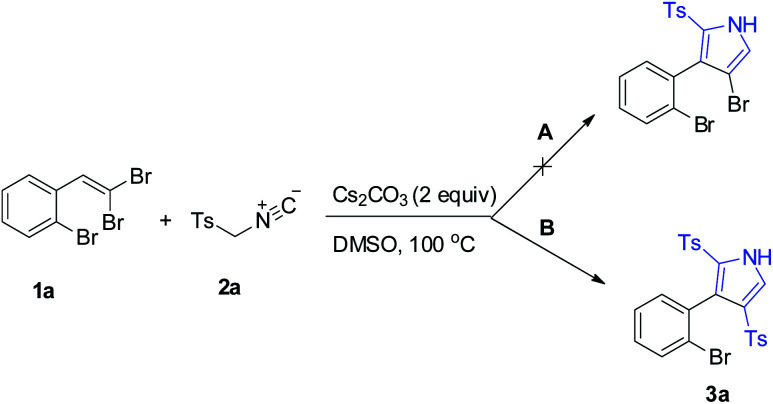
Formation of 2,4-ditosyl-1*H*-pyrrole instead of 4-bromo-2-tosyl-1*H*-pyrrole.

Inspired by our previously developed conditions,^17^ we started our survey by the cycloaddition of 1-bromo-2-(2,2-dibromovinyl)benzene (1a)^20^ and TosMIC (2a) under Cs_2_CO_3_ condition (2 equiv.) in DMSO at 100 °C. Two different routes were envisageable for the reaction (Scheme 2). What actually rationalizes the formation of 3a product (route B) is the occurrence of an additional step of the tosylation procedure, which affirms the dual role of TosMIC. Noteworthily, in this transformation, no by-product outcome from the direct [2 + 3]-cycloaddition of acetylene bromide (route A) was observed.

Our initial study focused on the exploration of optimal reaction conditions. As summarized in Table 1, to first evaluate the base efficiency, the reaction was conducted in a base-free media whereby no desired product was detected (Table 1, entry 1). This result encouraged us to screen numerous bases involving CsF, Cs_2_CO_3_, Et_3_N, K_2_CO_3_, Na_2_CO_3_, NaOAc, ^*t*^BuONa, and ^*t*^BuOK at 100 °C (Table 1, entries 2–9) and the best yield of 3a was obtained using Cs_2_CO_3_ in 78% yield (Table 1, entry 3). Notably, the counterion effect of the basic anions was compared, and the results showed its effective role in proceeding the reaction to high yields as the counterion size increased (Na, K to Cs). Also, when the amount of Cs_2_CO_3_ was decreased, the result reduced to 50% yield (Table 1, entry 10). Changing the reaction media to other solvents such as DMF, toluene or CH_3_CN, was not very successful to improve the product yield (Table 1, entries 11–13). In fact, the reaction failed in toluene (Table 1, entry 12). Implementing the reaction in ambient temperature offered no conversion, proving the distinct critical role of temperatures for the reaction to proceed (Table 1, entry 14). Summarizing the observations, we found that 2 equivalents of Cs_2_CO_3_ in DMSO at 100 °C were the best conditions listed in entry 3 and were chosen for the following explorations.

**Table 1:** Optimization of reaction condition^a^

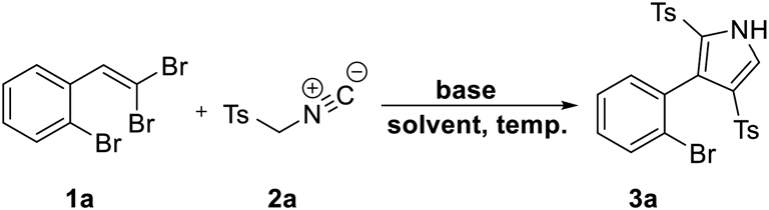				
Entry	Base (equiv.)	Solvent	Temp. (°C)	Yield^b^ (%)
1	—	DMSO	100	—
2	CsF (2)	DMSO	100	20
**3**	**Cs**_**2**_ **CO**_**3**_ **(2)**	**DMSO**	**100**	**78**
4	Et_3_N (2)	DMSO	100	Trace
5	K_2_CO_3_ (2)	DMSO	100	15
6	Na_2_CO_3_ (2)	DMSO	100	12
7	NaOAc (2)	DMSO	100	40
8	^*t*^BuONa (2)	DMSO	100	56
9	^*t*^BuOK (2)	DMSO	100	65
10	Cs_2_CO_3_ (1)	DMSO	100	50
11	Cs_2_CO_3_ (2)	DMF	100	75
12	Cs_2_CO_3_ (2)	Toluene	Reflux	Trace
13	Cs_2_CO_3_ (2)	CH_3_CN	Reflux	25
14	Cs_2_CO_3_ (2)	DMSO	rt	—

a Reaction conditions: 1a (1.0 mmol), 2a (2.0 mmol), base (2 equiv.), solvent (4.0 mL), temp. (100 °C) (bath temperature), 3–6 h.

b Isolated yields.

Under the determined optimal conditions, we perused the scope of reaction by numerous available starting materials. As illustrated in Table 2, the process was readily extended to a range of substituted aromatic and heteroaromatic *gem*-dibromoalkenes (1) with TosMIC (2a). The steric effects derived from *ortho*-substitutions and quinoline rings attached to the alkene had nearly no influence on these transformations (3a–3m). Similar to TosMIC, phenylsulfonylmethyl isocyanide (2b) also participated in the cascade reaction with 2,6-dichloro-3-(2,2-dibromovinyl)quinoline and 1-(2,2-dibromovinyl)-4-nitrobenzene without any difficulties, giving rise to the corresponding products in 80% and 87% yields, respectively (3n and 3o). Notably, the presence of a Me or an OMe group on the aryl ring of the *gem*-dibromoalkene did not afford the expected results.

**Table 2:** Scope of the synthesis of numerous 3-aryl-2,4-disulfonyl-1*H*-pyrroles 3^a^

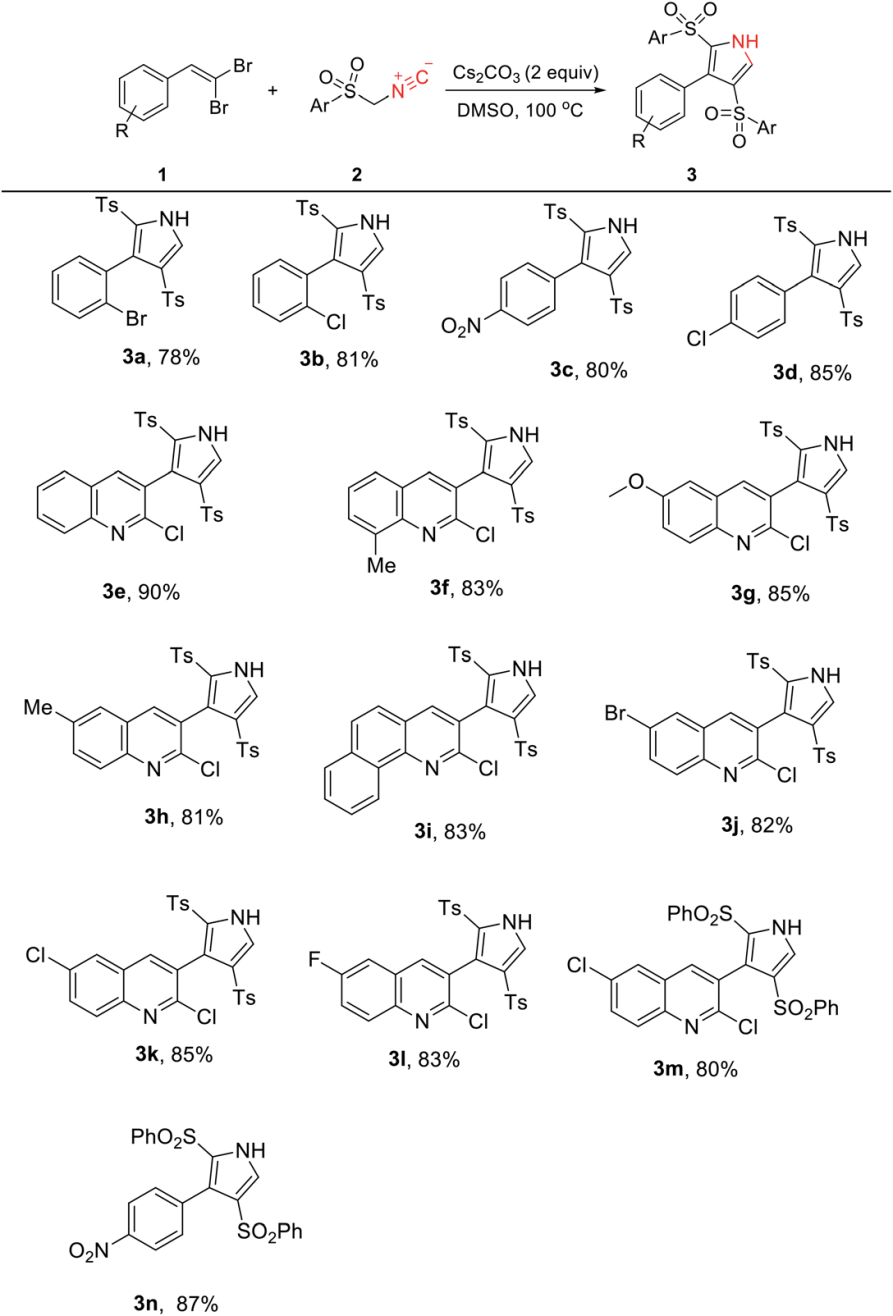

a Reaction conditions: 1 (1.0 mmol), 2 (2.0 mmol), Cs_2_CO_3_ (2 equiv.), DMSO (4.0 mL), temp. (100 °C) (bath temperature), 3–6 h.

**Scheme 3:**
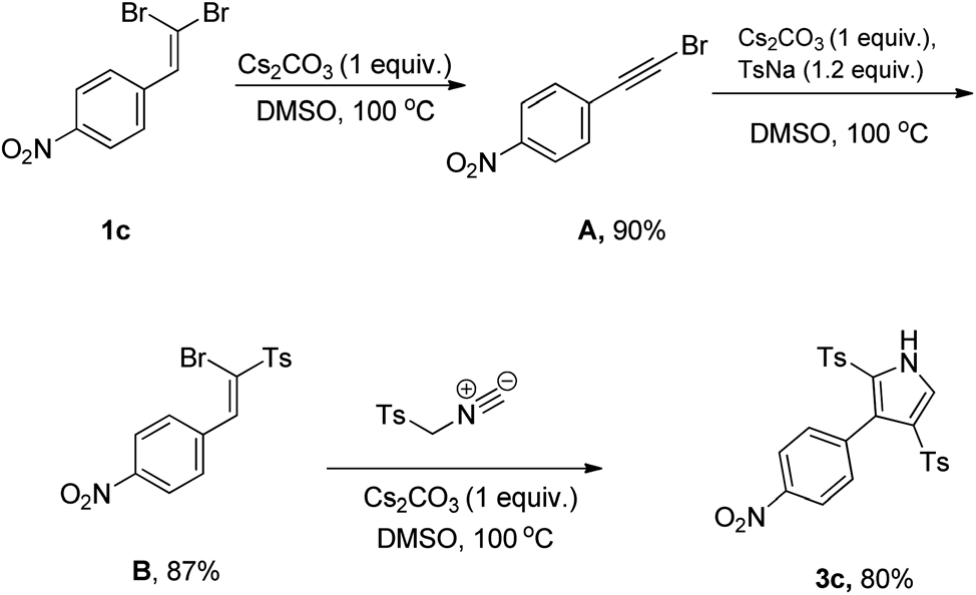
A designed sequences of control experiments for mechanistic investigation.

Further experiments were performed based on a designed series of control experiments to provide some insights into the reaction profile, as depicted in Scheme 3. Initially, the conversion of 1-(2,2-dibromovinyl)-4-nitrobenzene (1c) as the only starting material under the established condition was investigated, leading to the detection of desired acetylene bromide A. By assuming the following sulfonylation, we treated the resulting acetylene with sodium *p*-toluenesulfinate (TsNa) as the tosyl source based on our previously related study.^17^ Similarly, we found vinyl sulfone B as the only product, which in continuation, reacted with TosMIC in optimal conditions furnishing compound 3c in 80% yield.

**Scheme 4:**
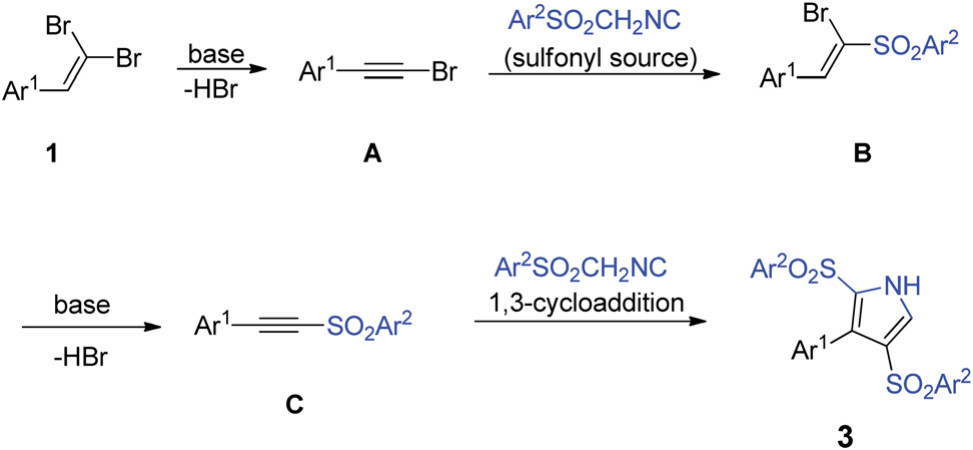
A plausible reaction mechanism.

Regarding the above-described findings, we suggested a plausible mechanism for this cascade reaction initiated by the HBr elimination process of 1,1-dibromoalkene 1 in an alkali media to form acetylene bromide A (Scheme 4). Subsequently, in a domino manner, TosMIC 2a acts as a sulfonyl source to render intermediate B, which undergoes the second HBr elimination to generate the sulfonyl acetylene C by Cs_2_CO_3_ assistance. Eventually, the [2 + 3]-cycloaddition of acetylene C with another TosMIC furnishes the desired product 3.

## Conclusions

We introduced a novel and straightforward strategy for the preparation of 3-aryl-2,4-disulfonyl-1*H*-pyrroles in high efficiency, which took advantage of a dual functional reactivity of arylsulfonyl methyl isocyanides against 1,1-dibromoalkenes under basic conditions. This process was facilitated in the presence of Cs_2_CO_3_, and its extensibility was evaluated for numerous starting materials. Furthermore, based on preliminary investigations of the reaction profile, a possible mechanism was proposed involving acetylenic intermediates. This report can be helpful to boost the practical developments of arylsulfonyl methyl isocyanides chemistry.

## Conflicts of interest

There are no conflict to declare.

## Supplementary Material

RA-011-D0RA10451E-s001
