# Supplementary material for: Cascade synthesis of 2,4-disulfonylpyrroles by the sulfonylation/[2 + 3]-cycloaddition reactions of gem-dibromoalkenes with arylsulfonyl methyl isocyanides
Source: RSC Adv. 2021 Apr 12;11(22):13292–6. doi: 10.1039/d0ra10451e (PMC8697567; doi:10.1039/d0ra10451e)
Supplement: RA-011-D0RA10451E-s001 [file RA-011-D0RA10451E-s001.pdf]

Electronic Supplementary Material (ESI) for RSC Advances.
This journal is © The Royal Society of Chemistry 2021

Supporting Information

Cascade Synthesis of 2,4-Disulfonylpyrroles by Sulfonylation/[2+3]-Cycloaddition Reactions of *gem*-Dibromoalkenes with Arylsulfonyl Methyl Isocyanides

Parvin Salehi,[a] Zahra Tanbakouchian,[a] Noushin Farajinia-Lehi,[a] and Morteza Shiri*[a]

[a] Department of Chemistry, Faculty of Physics and Chemistry, Alzahra University, Vanak, Tehran 1993893973, Iran, E-mail: mshiri@alzahra.ac.ir

Table of Contents

General information--**S02**

General procedures for the synthesis of 3-aryl-2,4-disulfonyl-1H-pyrroles --**S02**

Characterization of products--**S02**

NMR Spectra--**S10**

General Information

The solvents and chemicals were purchased from Merck and Aldrich chemical companies. Unless otherwise mentioned they were used without further purification. The 1,1-dibromoalkenes were prepared according to the reported procedures.[1] Melting points are taken on an Electrothermal 9100 apparatus and are uncorrected. FT-IR spectra were recorded on a Shimadzu Infra-Red Spectroscopy IR-435. Nuclear magnetic resonance (NMR) spectra were recorded on a Bruker AVANCE Spectrometer 400 MHz for 1H,100 MHz for 13C) in DMSO-d6 as solvent. Mass spectra recorded on Agilent Technology (HP) 5973 Network Mass Selective Detector operating at an ionization potential of 70 eV and a Leco CHNS, model 932 was used for elemental analysis.

General Procedure for the Synthesis of 2,4-Disulfonylpyrroles by Reaction of *gem*-Dibromoalkenes with Arylmethyl Sulfonyl Isocyanides

To a mixture of respective *gem*-dibromoalkene **1** (1.0 mmol) and Cs_2CO_3 (652 mg, 2.0 mmol) in DMSO (4.0 mL) was added the corresponding arylmethyl sulfonyl isocyanide (2.0 mmol). Then the mixture was stirred at 100 °C for 3-6 h. Upon completion of the reaction, H_2O (20 mL) was added and the whole was extracted with CH_2Cl_2 (20 mL). The organic layer was washed with brine and dried ($MgSO_4$). The solvent was removed and the residue was purified by column chromatography using *n*-hexane/EtOAc (6:4) to obtain **3** in pure form.

Characterization of Products

3-(2-Bromophenyl)-2,4-ditosyl-1H-pyrrole (3a)

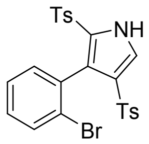

Yield: 412.6 mg (78%). White solid. M.p. = 105-107 ^{o}C. **FT-IR** (KBr): 3415, 3060, 2023, 2854, 1645, 1595, 1380, 1146 cm^{-1}.

^{1}H NMR (400 MHz, DMSO-d_6) δ = 13.48 (s, 1H), 7.90 (s, 1H), 7.56-7.54 (m, 1H), 7.47 (d, J = 8.0 Hz, 2H), 7.35 (d, J = 8.0 Hz, 4H), 7.26 (q, 4H), 6.82 (t, J = 4.4 Hz, 1H), 2.36 (s, 3H), 2.34 (s, 3H) ppm. **^{13}C NMR** (100 MHz, DMSO-d_6) δ = 145.0, 144.1, 139.3, 138.3, 132.7,

132.1, 131.5, 130.6, 130.3, 129.9, 128.5, 127.7, 127.5, 126.8, 126.2, 126.0, 125.3, 21.5, 21.4 ppm. **MS (EI)** m/z: calcd for $C_{24}H_{20}BrNO_4S_2$; 529.0, found, 530.45. **Anal. calcd**. For $C_{24}H_{20}BrNO_4S_2$; C, 54.34; H, 3.80; N, 2.64; S, 12.09%; Found; C, 54.43; H, 3.75; N, 2.60; S, 12.00%.

3-(2-Chlorophenyl)-2,4-ditosyl-1H-pyrrole (3b)

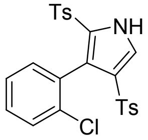

Yield: 392.8 mg (81%). White solid. M.p. = 131-134 °C. **FT-IR** (KBr): 3298, 2956, 1654, 1517, 1343, 1143 cm^{-1}. **^{1}H NMR** (400 MHz, DMSO-*d*$_6$) δ = 13.46 (s, 1H), 7.87 (s, 1H), 7.44 (q, 3H), 7.35 (t, J = 7.0 Hz, 3H), 7.31-7.22 (m, 5H), 6.84 (d, J = 7.6 Hz, 1H), 2.36 (s, 3H), 2.34 (s, 3H) ppm. **^{13}C NMR** (100 MHz, DMSO-*d*$_6$) δ = 144.8, 144.0, 139.4, 138.5, 135.1, 132.9, 130.4, 130.3, 129.9, 129.7, 129.0, 128.2, 127.5, 127.4, 126.2, 125.3, 124.4, 21.5, 21.4 ppm. **MS (EI)** m/z: calcd for $C_{24}H_{20}ClNO_4S_2$; 485.05, found, 486.00. **Anal. calcd**. For $C_{24}H_{20}ClNO_4S_2$; C, 59.31; H, 4.15; N, 2.88; S, 13.19%; Found; C, 59.48; H, 4.25; N, 2.86; S, 13.00%.

3-(4-Nitrophenyl)-2,4-ditosyl-1H-pyrrole (3c)

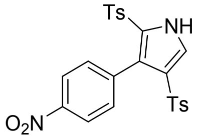

Yield 396.8 mg (80%). White solid. M.p. = 185-187 °C. **FT-IR** (KBr): 3444, 3063, 2923, 2854, 1645, 1596, 1383, 1147 cm^{-1}.

^{1}H NMR (400 MHz, DMSO-*d*$_6$) δ = 13.68 (s, 1H), 8.18 (d, J = 8.4 Hz, 2H), 7.96 (s, 1H), 7.46 (d, J = 8.0 Hz, 2H), 7.36 (d, J = 8.0 Hz, 2H), 7.29 (d, J = 8.9 Hz, 2H), 7.24 (d, J = 8.0 Hz, 2H), 7.10 (d, J = 8.4 Hz, 2H), 2.36 (s, 3H), 2.33 (s, 3H) ppm. **^{13}C NMR** (100 MHz, DMSO-*d*$_6$) δ = 147.7, 145.3, 144.4, 139.2, 138.3, 137.4, 132.5, 130.6, 130.2, 129.1, 128.1, 127.3, 125.9, 125.3, 122.7, 21.5, 21.4 ppm. **MS (EI)** m/z: calcd for $C_{24}H_{20}N_2O_6S_2$; 496.08, found, 496.55. **Anal. calcd**. For $C_{24}H_{20}N_2O_6S_2$; C, 58.05; H, 4.06; N, 5.64; S, 12.91%; Found; C, 58.13; H, 4.00; N, 5.60; S, 13.00%.

3-(4-Chlorophenyl)-2,4-ditosyl-1H-pyrrole (3d)

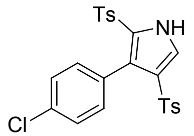

Yield: 412 mg (85%). White solid. M.p. = 150-153 ^oC. **FT-IR** (KBr): 3328, 2950, 1554, 1517, 1243, 1143, 870 cm^{-1}. **^1H NMR** (300 MHz, DMSO-d_6) δ = 13.61 (s, 1H), 8.20 (d, J = 8.7 Hz, 2H), 7.90 (d, J = 8.4 Hz, 2H), 7.49 (q, 4H), 7.38 (t, J = 7.05 Hz, 3H), 7.28 (d, J = 8.4 Hz, 2H), 2.45 (s, 3H), 2.35 (s, 3H) ppm. **^{13}C NMR** (75 MHz, DMSO-d_6) δ = 147.3, 145.1, 144.5, 139.9, 139.8, 138.4, 132.1, 131.2, 130.2, 130.0, 128.5, 127.2, 126.4, 124.5, 123.90, 123.1, 21.6, 21.5 ppm. **MS (EI)** m/z: calcd for $C_{24}H_{20}ClNO_4S_2$; 485.05, found, 486.00. **Anal. calcd**. For $C_{24}H_{20}ClNO_4S_2$; C, 59.31; H, 4.15; N, 2.88; S, 13.19%; Found; C, 59.53; H, 4.19; N, 2.80; S, 12.99%.

2-Chloro-3-(2,4-ditosyl-1H-pyrrol-3-yl)quinolone (3e)

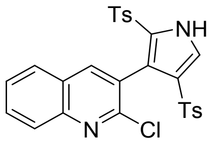

Yield: 483.3 mg (90%). White solid. M.p. = 106-109 ^oC. **FT-IR** (KBr): 3383, 3062, 2924, 1322, 1146 cm^{-1}. **^1H NMR** (500 MHz, $CDCl_3$) δ = 11.00 (s, 1H), 8.10 (t, J = 9.75 Hz, 1H), 7.85-7.72 (m, 4H), 7.64 (q, 1H), 7.32 (d, J = 8.0 Hz, 2H), 7.26 (t, J = 8.0 Hz, 2H), 7.07 (d, J = 8.0 Hz, 2H), 7.03 (d, J = 7.5 Hz, 2H), 2.32 (s, 6H) ppm. **^{13}C NMR** (125 MHz, $CDCl_3$) δ = 150.7, 147.4, 145.2 ,144.3 ,143.9 ,141.8 , 141.0, 138.0, 136.8, 131.2, 129.8, 129.5, 128.3, 128.0, 127.8, 127.7, 127.6, 127.5, 127.3, 126.1, 125.4, 122.6, 106.9, 106.8, 106.7, 21.6, 21.5 ppm. **MS (EI)** m/z: calcd for $C_{27}H_{21}ClN_2O_4S_2$; 536.06, found, 537.05. **Anal. calcd**. For $C_{27}H_{21}ClN_2O_4S_2$; C, 60.39; H, 3.94; N, 5.22; S, 11.94%; Found; C, 60.51; H, 4.15; N, 5.28; S, 12.09%.

2-Chloro-3-(2,4-ditosyl-1H-pyrrol-3-yl)-8-methylquinoline (3f)

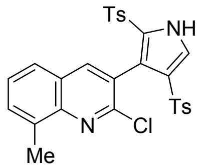

Yield: 458.2 mg (83%). White solid. M.p. = 107-110 °C. **FT-IR** (KBr): 3274, 3062, 2922, 1311, 1144 cm^{-1}. **^{1}H NMR** (400 MHz, DMSO-d_6) δ = 13.62 (s, 1H), 8.17(s, 1H), 7.89 (d, J = 8.4 Hz, 2H), 7.81 (d, J = 8 Hz, 1H), 7.73 (d, J = 6.8 Hz, 1H), 7.62-7.56 (m, 2H), 7.50 (d, J = 8.4 Hz, 2H), 7.44 (s, 1H), 7.38 (d, J = 8.4 Hz, 2H), 7.27 (d, J = 8 Hz, 2H), 2.70 (s, 3H), 2.45 (s, 3H), 2.36 (s, 3H) ppm. **^{13}C NMR** (100 MHz, DMSO-d_6) δ = 150.2, 146.0, 145.2, 144.5, 141.8, 139.7, 138.4, 135.9, 131.5, 131.1, 130.3, 129.8, 128.3, 127.7, 127.5, 126.7, 126.4, 126.1, 125.0, 124.9, 123.5, 21.6, 21.5, 17.9 ppm. **MS (EI)** m/z: calcd for $C_{28}H_{23}ClN_2O_4S_2$; 551.07, found, 552.08. **Anal. calcd**. For $C_{28}H_{23}ClN_2O_4S_2$; C, 61.03; H, 4.21; N, 5.08; S, 11.64%; Found; C, 60.99; H, 4.15; N, 5.13; S, 11.70%.

2-Chloro-3-(2,4-ditosyl-1H-pyrrol-3-yl)-6-methoxyquinoline (3g)

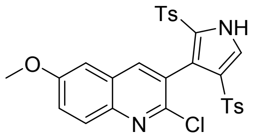

Yield: 481 mg (85%). White solid. M.p. = 121-124 °C. **FT-IR** (KBr): 3422, 3061, 2924, 1313, 1140 cm^{-1}. **^{1}H NMR** (400 MHz, DMSO-d_6) δ = 13.59 (s, 1H), 8.04 (d, J = 16.4 Hz, 1H), 7.94-7.88 (m, 3H), 7.50 (d, J = 8.4 Hz, 3H), 7.43 (d, J = 4.4 Hz, 1H), 7.35 (q, 3H), 7.27 (d, J = 7.2 Hz, 1H), 3.92 (s, 3H), 2.46 (s, 3H), 2.36 (s, 3H) ppm. **^{13}C NMR** (100 MHz, DMSO-d_6) δ = 158.4, 158.3, 148.4, 145.1, 144.4, 143.5, 142.8, 140.4, 139.7, 138.5, 130.3, 129.9, 129.6, 128.3, 127.9, 127.6, 127.5, 125.0, 123.8, 123.5 106.4, 56.2, 21.6, 21.5 ppm. **MS (EI)** m/z: calcd for $C_{28}H_{23}ClN_2O_5S_2$; 566.07, found, 567.07. **Anal. calcd**. For $C_{28}H_{23}ClN_2O_5S_2$; C, 59.31; H, 4.09; N, 4.94; S, 11.31%; Found; C, 60.01; H, 4.15; N, 4.95; S, 11.25%.

2-Chloro-3-(2,4-ditosyl-1H-pyrrol-3-yl)-6-methylquinoline (3h)

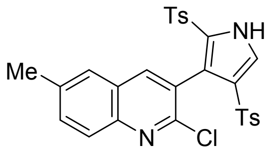

Yield: 446.3 mg (81%). White solid. M.p. =103-106 ^{o}C. **FT-IR** (KBr): 3446, 3060, 1329, 1145 cm^{-1}. **^{1}H NMR** (400 MHz, DMSO-d_6) δ = 13.70 (s, 1H), 8.03 (d, J = 4.8 Hz, 1H), 7.95 (q, 1H), 7.81 (s, 1H), 7.74 (t, J = 7.0 Hz, 2H), 7.51 (q, 3H), 7.34 (d, J = 8.0 Hz, 2H), 7.28 (d, J = 8.0 Hz, 2H), 7.20 (d, J = 8.0 Hz, 2H), 2.57 (s, 3H), 2.34 (s, 3H), 2.32 (s, 3H) ppm. **^{13}C NMR** (100 MHz, DMSO-d_6) δ = 149.7, 145.2, 144.3, 140.6, 139.3, 138.1, 137.7, 133.8, 130.5, 130.1, 129.2, 128.2, 127.9, 127.6, 127.5, 127.4, 127.30, 126.4, 125.7, 123.9, 122.7, 21.6, 21.5, 21.4 ppm. **MS (EI)** m/z: calcd for $C_{28}H_{23}ClN_2O_4S_2$; 550.08, found, 551.07. **Anal. calcd**. For $C_{28}H_{23}ClN_2O_4S_2$; C, 61.03; H, 4.21; N, 5.08; S, 11.64%; Found; C, 60.98; H, 4.25; N, 5.10; S, 11.70%.

2-Chloro-3-(2,4-ditosyl-1H-pyrrol-3-yl)benzo[h]quinolone (3i)

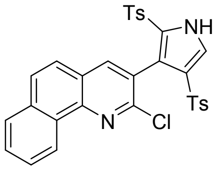

Yield: 487 mg (83%). White solid. M.p. = 161-164 ^{o}C. **FT-IR** (KBr): 3436, 3055, 2922, 1321, 1146 cm^{-1}. **^{1}H NMR** (400 MHz, DMSO-d_6) δ = 13.65 (s, 1H), 9.06-9.03 (m, 1H), 8.28 (s, 1H), 8.12 (q, 1H), 8.07 (d, J = 8.8 Hz, 1H), 7.90 (t, J = 8.6 Hz, 3H), 7.84 (q, 2H), 7.51 (d, J = 10.0 Hz, 3H), 7.38 (d, J = 8.0 Hz, 2H), 7.26 (d, J = 8.0 Hz, 2H), 2.46 (s, 3H), 2.35 (s, 3H) ppm. **^{13}C NMR** (100 MHz, DMSO-d_6) δ = 162.9, 150.3, 145.3, 145.2, 144.5, 141.8, 139.6, 138.4, 134.1, 130.3, 129.9, 129.8, 129.6, 128.8, 128.7, 128.3, 128.2, 127.5, 127.0, 125.3, 125.0, 124.9, 124.8, 124.2, 123.4, 21.6, 21.5 ppm. **MS (EI)** m/z: calcd for $C_{31}H_{23}ClN_2O_4S_2$; 586.08, found, 587.11. **Anal. calcd**. For $C_{31}H_{23}ClN_2O_4S_2$; C, 63.42; H, 3.95; N, 4.77; S, 10.92%; Found; C, 63.49; H, 4.02; N, 4.56; S, 10.88%.

6-Bromo-2-chloro-3-(2,4-ditosyl-1H-pyrrol-3-yl)quinolone (3j)

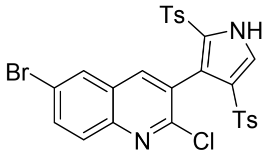

Yield 503 mg (82%). White solid. M.p. = 116-119 °C. **FT-IR** (KBr): 3423, 3106, 2924, 1332, 1154 cm^{-1}. **^{1}H NMR** (400 MHz, DMSO-d_6) δ = 13.77 (S, 1H), 8.16 (q, 1H), 8.11-8.06 (m, 2H), 7.97-7.89 (m, 2H), 7.52 (q, 2H), 7.35 (d, J = 8.4 Hz, 2H), 7.30 (t, J = 4.0 Hz, 2H), 7.21 (d, J = 8.0 Hz, 2H), 2.35 (s, 3H), 2.33 (s, 3H) ppm. **^{13}C NMR** (100 MHz, DMSO-d_6) δ = 151.2, 145.9, 145.5, 145.3, 145.0, 144.4, 140.5, 139.5, 139.2, 137.9, 132.4, 132.3, 130.6, 130.2, 129.3, 128.4, 128.3, 127.6, 127.5, 127.4, 127.3, 125.7, 125.3, 123.6, 122.1, 21.5, 21.4 ppm. **MS (EI)** m/z: calcd for $C_{27}H_{20}BrClN_2O_4S_2$; 613.97, found, 615.94. **Anal. calcd**. For $C_{27}H_{20}ClN_2O_4S_2$; C, 52.65; H, 3.27; N, 4.55; S, 10.41%; Found; C, 52.55; H, 3.34; N, 4.51; S, 10.12%.

2,6-Dichloro-3-(2,4-ditosyl-1H-pyrrol-3-yl)quinolone (3k)

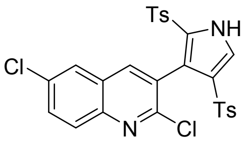

Yield: 485.7 mg (85%). White solid. M.p. = 134-137 °C. **FT-IR** (KBr): 3435, 3135, 3064, 2926, 1319, 1170 cm^{-1}. **^{1}H NMR** (400 MHz, DMSO-d_6) δ = 13.65 (s, 1H), 8.25 (s, 1H), 8.14 (t, J = 2.2 Hz, 1H), 8.05 (q, 1H), 7.91-7.87 (m, 4H), 7.50 (d, J = 8.0 Hz, 2H), 7.46 (d, J = 3.2 Hz, 1H), 7.37 (q, 2H), 7.27 (d, J = 7.6 Hz, 1H), 2.45 (s, 3H), 2.36 (s, 3H) ppm. **^{13}C NMR** (100 MHz, DMSO-d_6) δ = 151.8, 145.8, 145.7, 145.3, 145.2, 144.5, 140.7, 139.6, 138.4, 132.4, 132.3, 132.1, 130.3, 129.9, 128.3, 127.6, 127.5, 127.1, 125.1, 124.9, 123.0, 21.6, 21.5 ppm. **MS (EI)** m/z: calcd for $C_{27}H_{20}Cl_2N_2O_4S_2$; 570.02, found, 571.49. **Anal. calcd**. For $C_{27}H_{20}Cl_2N_2O_4S_2$; C, 56.75; H, 3.53; N, 4.90; S, 11.22%; Found; C, 57.01; H, 3.45; N, 4.85; S, 11.18%.

2-Chloro-3-(2,4-ditosyl-1H-pyrrol-3-yl)-6-fluoroquinoline (3l)

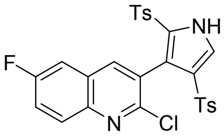

Yield: 459.8 mg (83%). White solid. M.p. = 144-147 °C. **FT-IR** (KBr): 3345, 3125, 3046, 2962, 1319, 1170 cm^{-1}. **^{1}H NMR** (300 MHz, DMSO-d_6) δ = 13.74 (s, 1H), 8.06 (s, 1H), 8.94 (s, 1H), 7.85 (d, *J* = 9 Hz, 2H), 7.51 (d, *J* = 7.8 Hz, 2H), 7.50 (d, *J* = 8.0 Hz, 2H), 7.33 (q, *J* = 7.6 Hz, 5H), 7.22 (d, *J* = 7.8 Hz, 2H), 2.35 (s, 3H), 2.33 (s, 3H) ppm. **^{13}C NMR** (75 MHz, DMSO-d_6) δ = 150.2, 145.3, 144.4, 144.2,139.2,

138.0, 131.1, 131.0, 130.5, 130.2, 129.3, 128.3, 127.5, 127.3, 125.7, 125.1, 122.2, 121.9, 121.5, 21.5, 21.4 ppm. **MS (EI)** m/z: calcd for $C_{27}H_{20}ClFN_2O_4S_2$; 554.05, found, 555.04. **Anal. calcd**. For $C_{27}H_{20}ClFN_2O_4S_2$; C, 58.43; H, 3.63; N, 5.05; S, 11.55%; Found; C, 58.21; H, 3.57; N, 4.89; S, 11.38%.

3-(2,5-Bis(phenylsulfonyl)-1H-pyrrol-3-yl)-2,6-dichloroquinoline (3m)

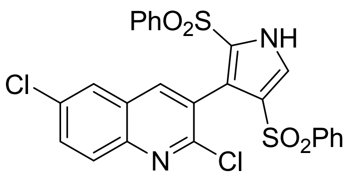

Yield: 434.7 mg (80%). White solid. M.p. = 148-151 ^oC. **FT-IR** (KBr): 3335, 3085, 2915, 1328, 1172 cm^{-1}. **^{1}H NMR** (300 MHz, DMSO-d_6) δ = 13.75 (s, 1H), 8.28 (d, J = 13.5 Hz, 1H), 8.15 (s, 1H), 8.08-8.00 (m, 3H), 7.90 (dd, J = 2.1 Hz, J = 8.85 Hz, 1H), 7.82-7.63 (m, 4H), 7.5 (q, 51H) ppm. **^{13}C NMR** (75 MHz, DMSO-d_6) δ = 151.7, 145.9, 145.4, 142.4, 141.3, 140.8, 140.1, 134.5, 134.0, 132.3, 132.1, 130.3, 129.9, 129.5, 128.2, 127.4, 127.3, 127.2, 125.4, 124.7, 123.2 ppm. **MS (EI)** m/z: calcd for $C_{25}H_{16}Cl_2N_2O_4S_2$; 541.99, found, 543.43. **Anal. calcd**. For $C_{25}H_{16}Cl_2N_2O_4S_2$; C, 55.26; H, 2.97; N, 5.16; S, 11.80%; Found; C, 55.38; H, 3.13; N, 5.04; S, 11.77%.

3-(4-Nitrophenyl)-2,4-bis(phenylsulfonyl)-1H-pyrrole (3n)

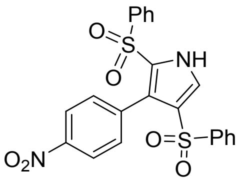

Yield: 408 mg (87%). White solid. M.p. = 183-186 ^oC. **FT-IR** (KBr): 3446, 3053, 2922, 2855, 1650, 1586, 1373, 1174 cm^{-1}. **^{1}H NMR** (300 MHz, DMSO-d_6) δ = 13.76 (s, 1H), 8.16 (d, J = 8.7 Hz, 2H), 8.01 (s, 1H), 7.57 (q, 4H), 7.48-7.40 (m, 4H), 7.09 (d, J = 8.7 Hz, 2H) ppm. **^{13}C NMR** (75 MHz, DMSO-d_6) δ = 147.8, 141.9, 141.2, 137.2, 134.5, 133.9, 132.5, 130.2, 129.7, 128.9, 128.4, 127.2, 125.7, 125.6, 122.7

ppm. **MS (EI)** m/z: calcd for $C_{22}H_{16}N_2O_6S_2$; 468.04, found, 468.50. **Anal. calcd**. For $C_{22}H_{16}N_2O_6S_2$; C, 56.40; H, 3.44; N, 5.98; S, 13.69%; Found; 56.38; H, 3.52; N, 5.84; S, 13.74%.

References:

1 (a) N. B. Desai, N. McKelvie and F. Ramirez, A new synthesis of 1, 1-dibromoölefins via phosphine-dibromomethylenes. The reaction of triphenylphosphine with carbon tetrabromide. *J. Am. Chem. Soc.* 1962, **84**, 1745; (b) D. Nandini, M. Asthana, K. Mishra, R. P. Singh and R. M. Singh, Temperature dependent selective synthesis of linear 2-bromo and 2-alkoxyfuro [2, 3-b] quinolines: reaction of 3-(2, 2-dibromovinyl-) quinolin-2 (1H)-ones with alcoholic KOH. *Tetrahedron Lett.* 2014, **55**, 6257.

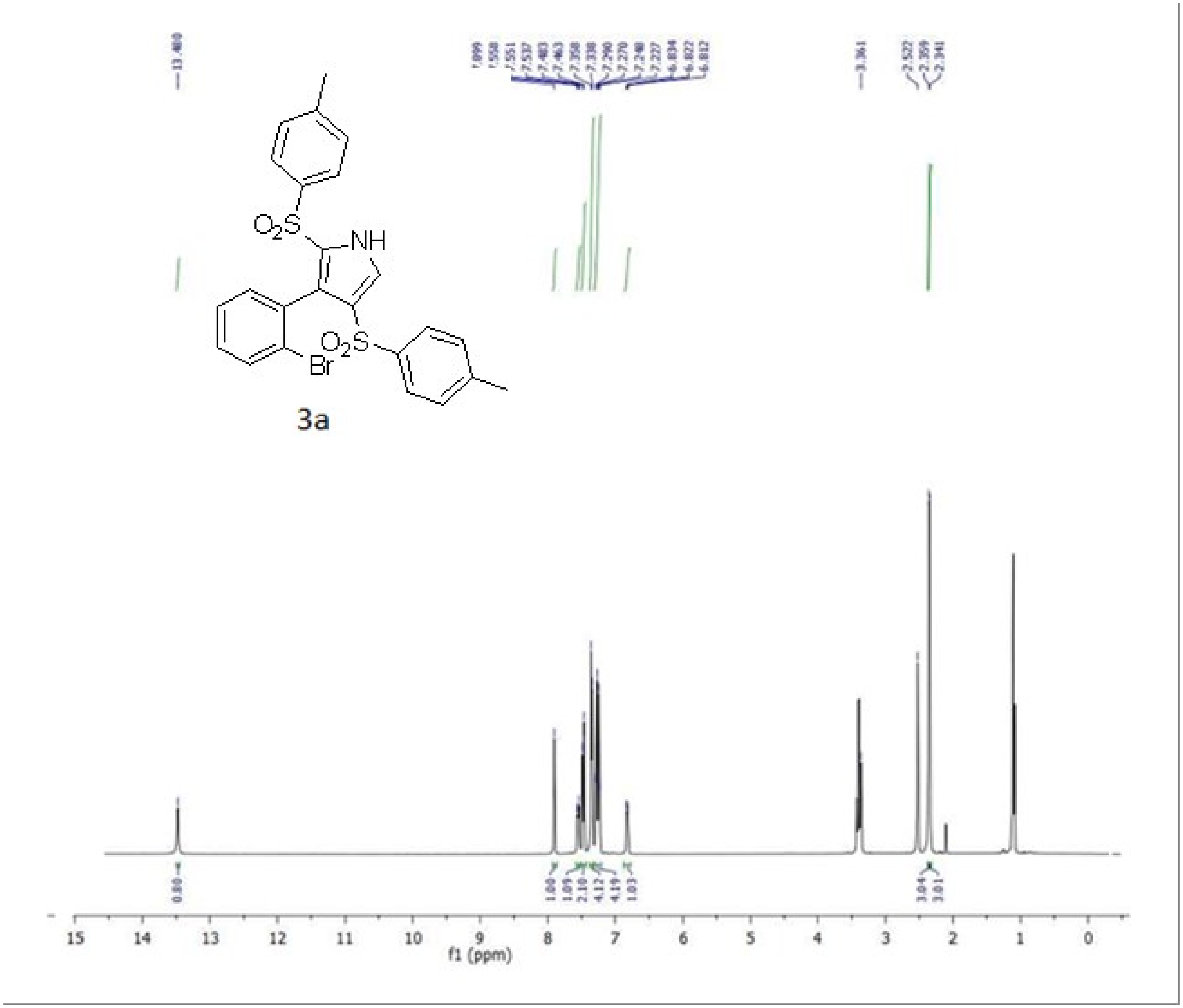

13.480
3.361
2.522
2.359
2.341
O2S
NH
Br
3a
0.80
1.00
1.09
2.10
4.12
4.19
1.03
3.04
3.01
15 14 13 12 11 10 9 8 7 6 5 4 3 2 1 0
f1 (ppm)

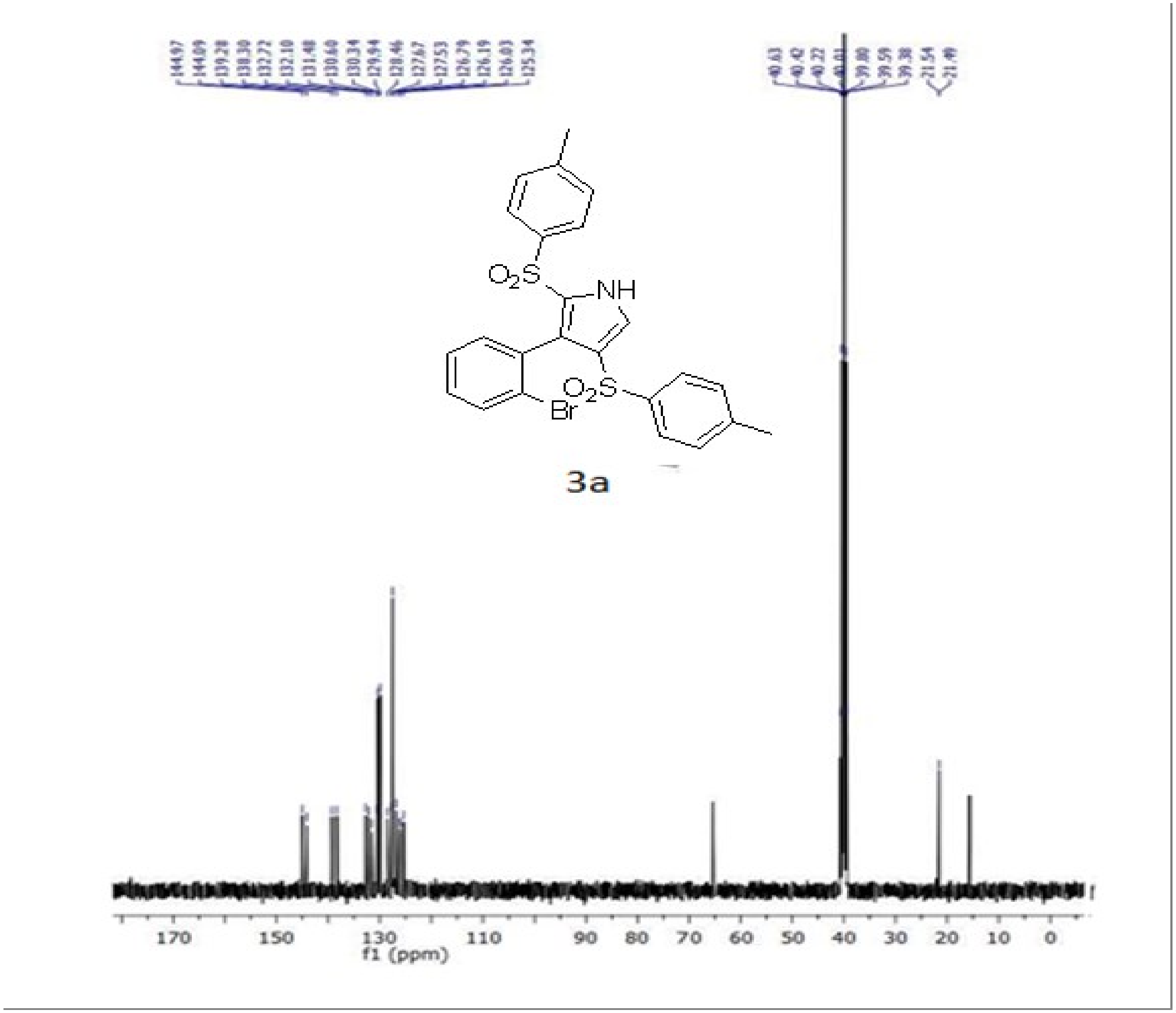
144.97
144.09
139.28
138.30
132.72
132.10
131.48
130.60
130.34
129.94
128.46
127.67
127.53
126.79
126.19
126.03
125.34
40.63
40.42
40.22
40.01
39.80
39.59
39.38
21.54
21.49
O2S
NH
O2S
Br
3a
170
150
130
110
90
80
70
60
50
40
30
20
10
0
f1 (ppm)

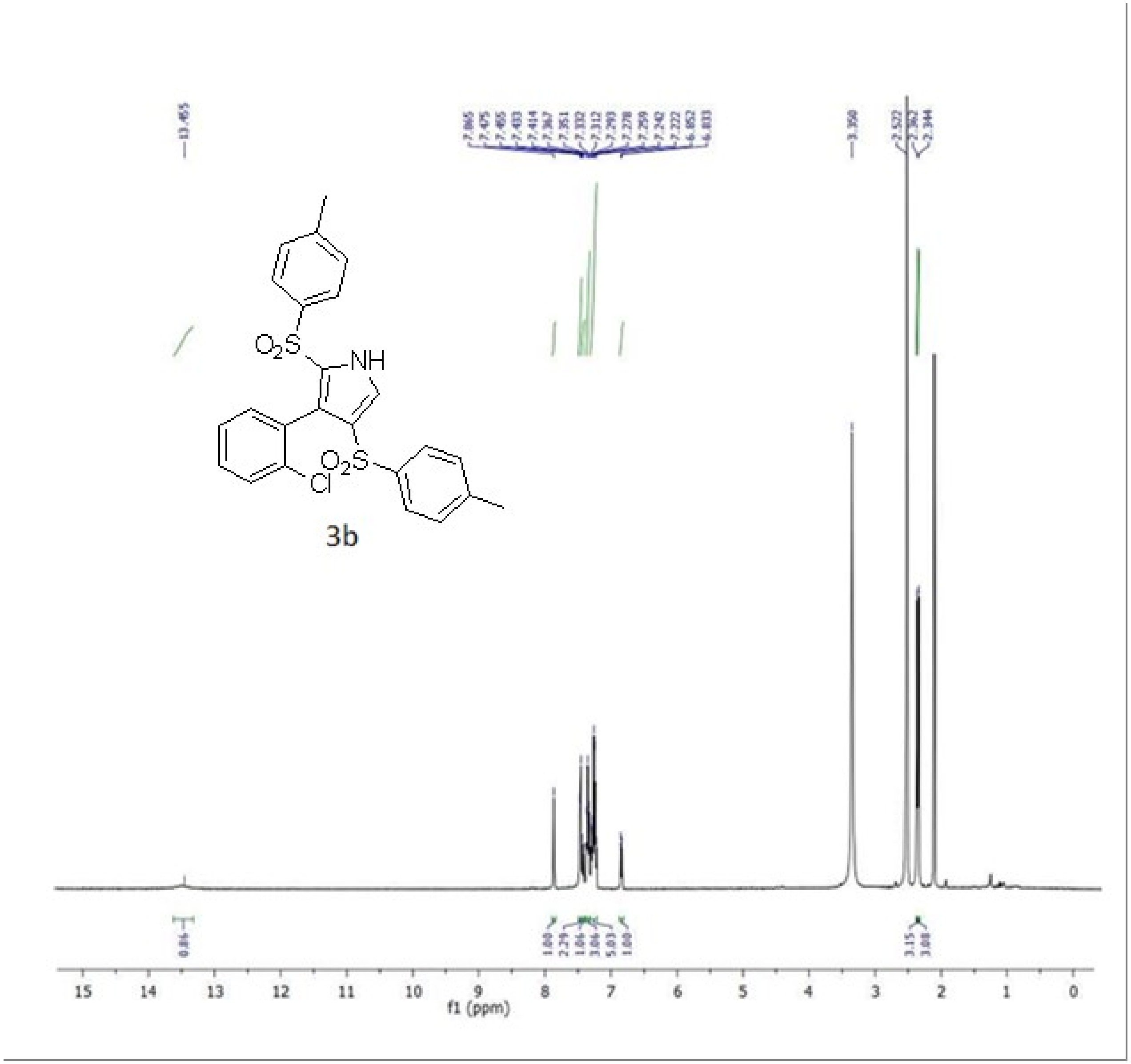

O2S
NH
O2S
Cl
3b
f1 (ppm)

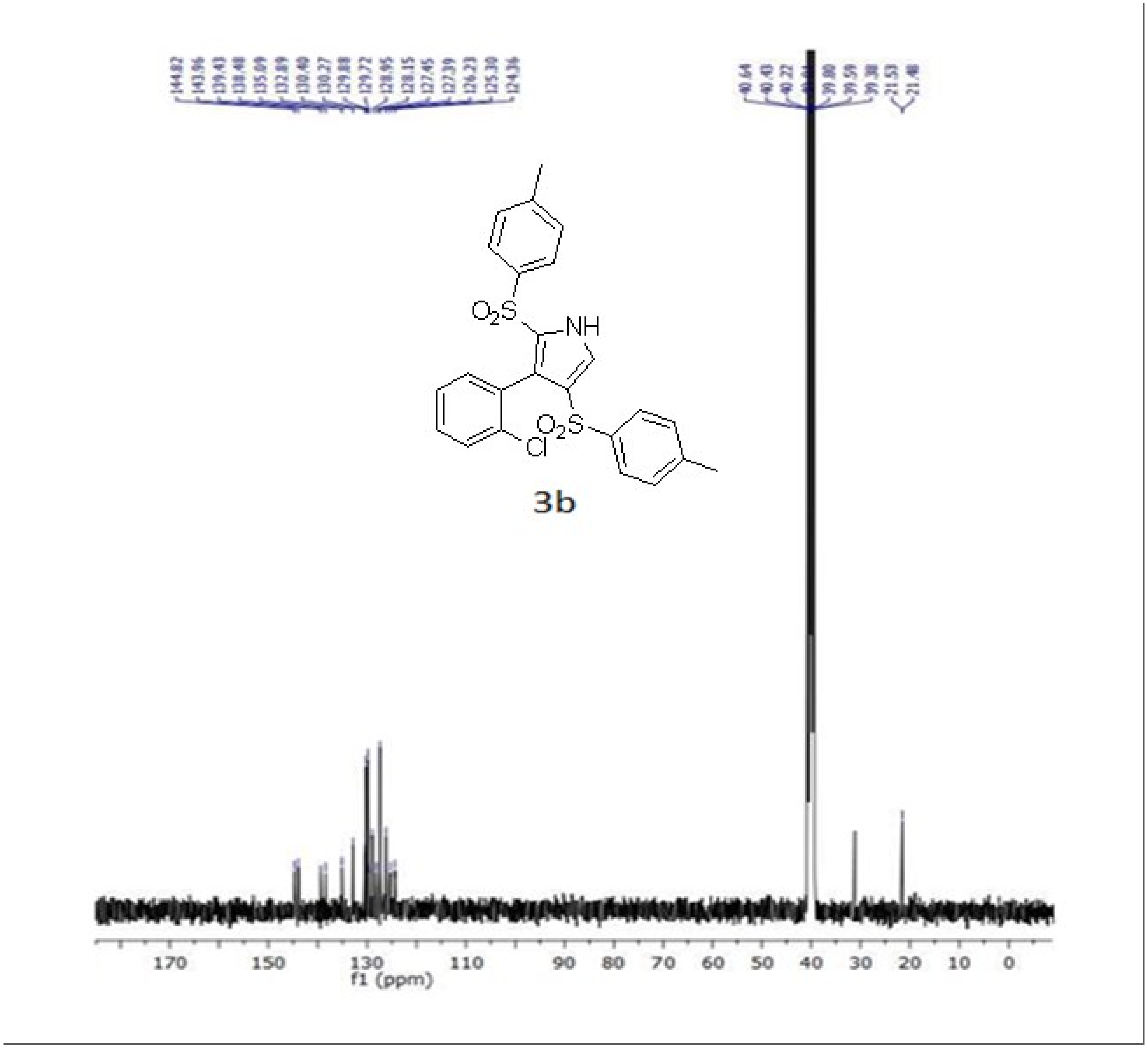
144.82
143.96
139.43
138.48
135.09
132.89
130.40
130.27
129.88
129.72
128.95
128.15
127.45
127.39
126.23
125.30
124.36
40.64
40.43
40.22
39.80
39.59
39.38
21.53
21.48
O2S
NH
O2S
Cl
3b
170
150
130
110
90
80
70
60
50
40
30
20
10
0
f1 (ppm)

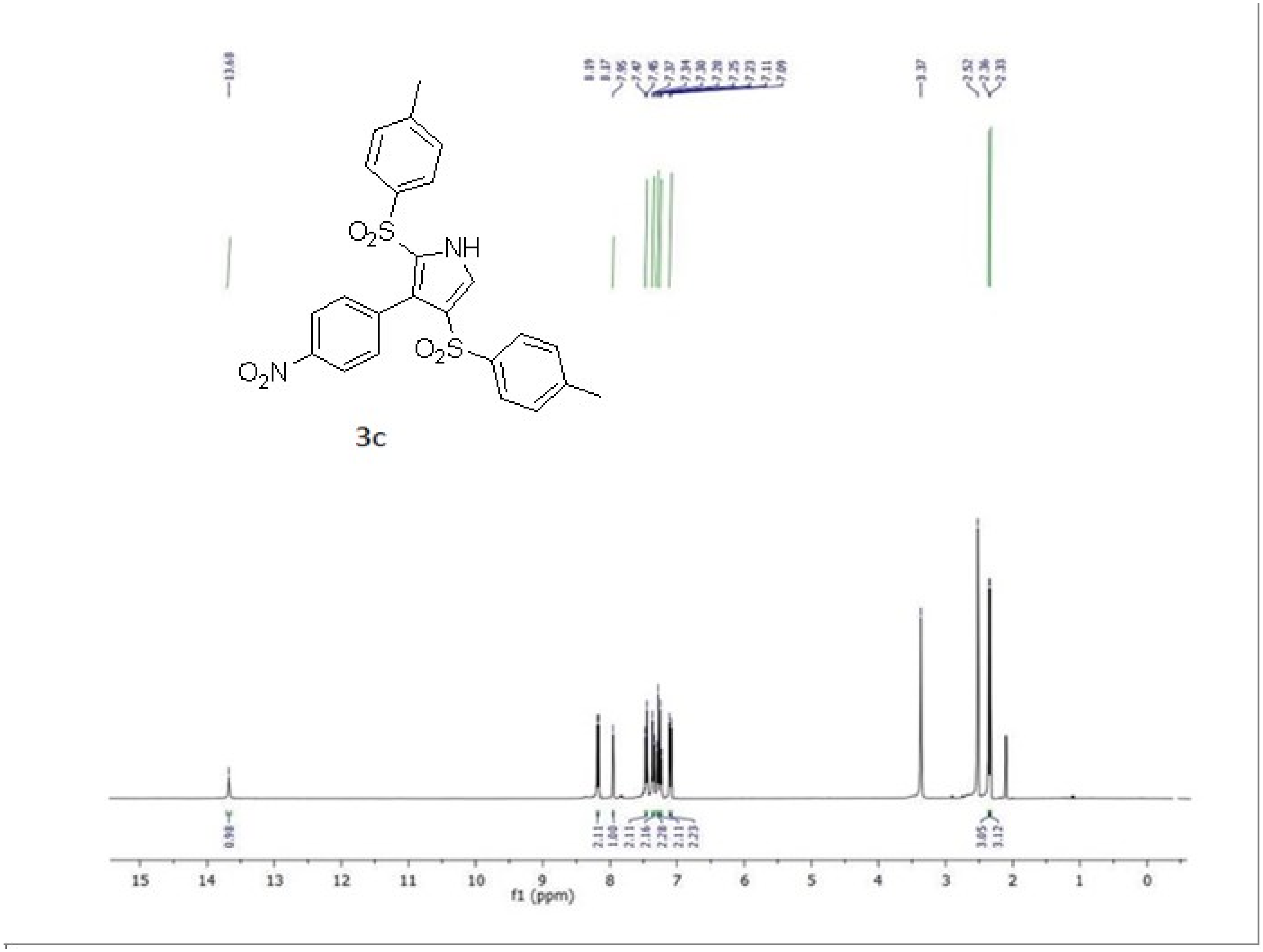

3c
f1 (ppm)

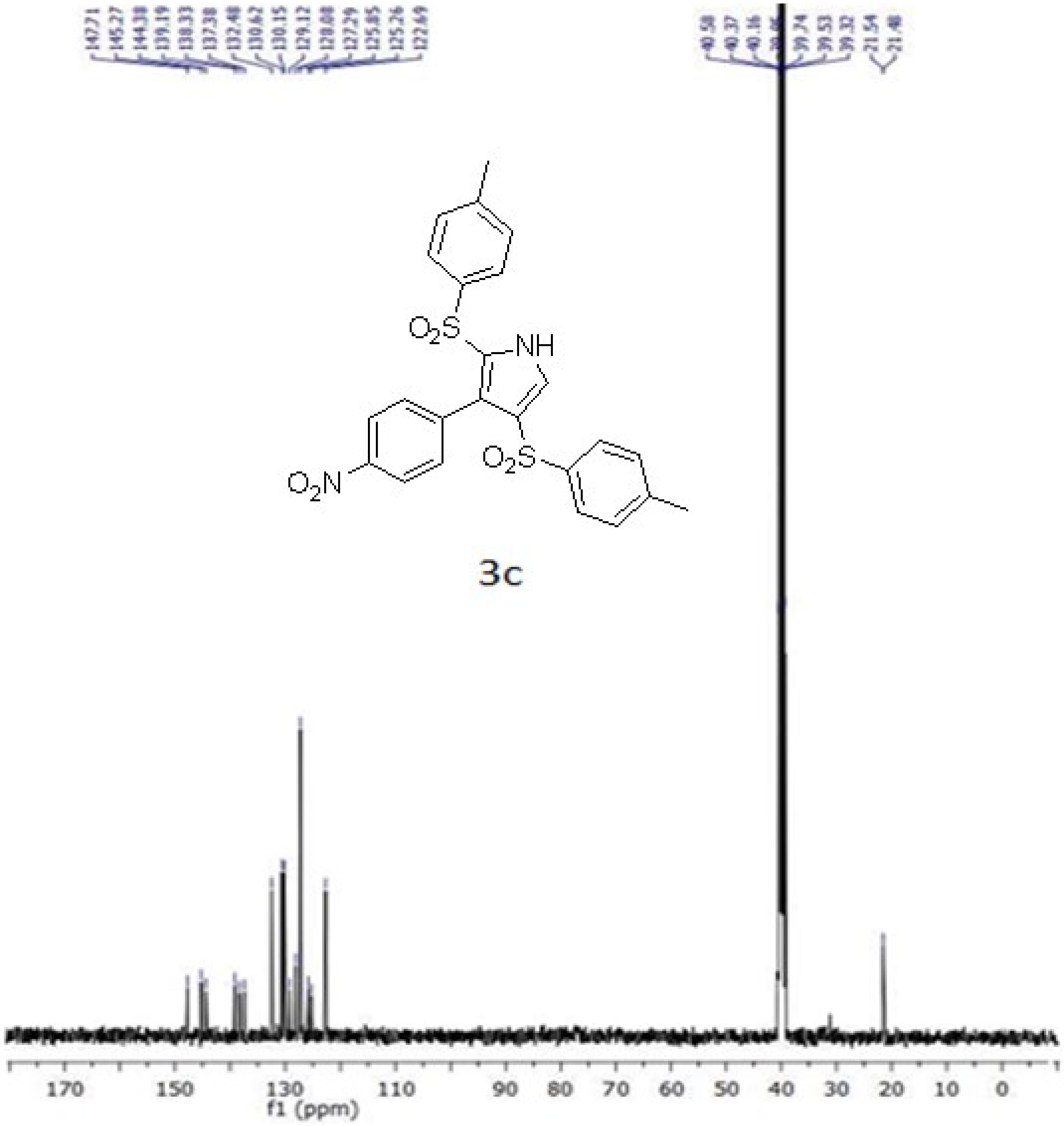
O2S
NH
O2S
O2N
3c
170 150 130 110 90 80 70 60 50 40 30 20 10 0
f1 (ppm)

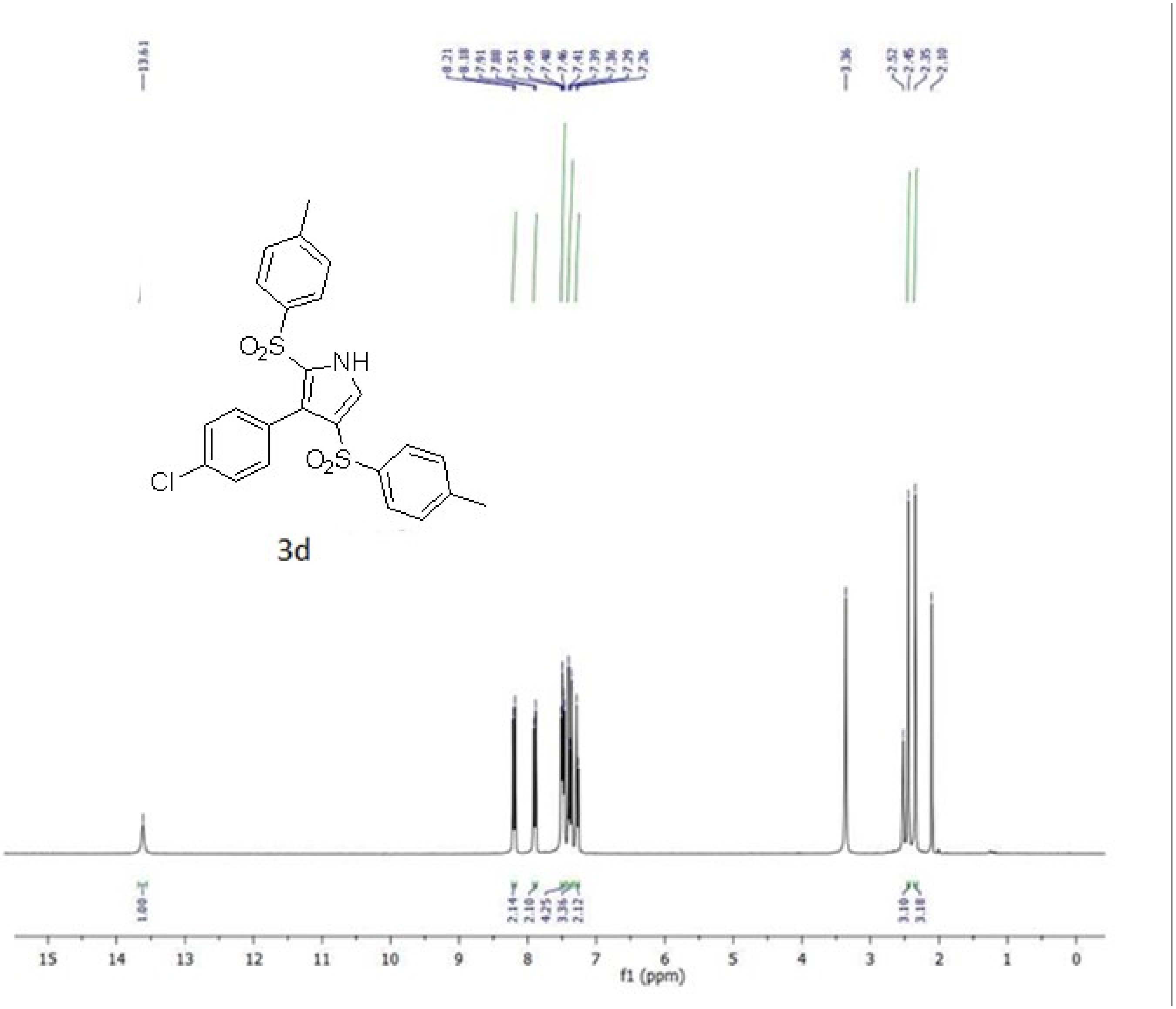

3d
O2S
NH
Cl
O2S
f1 (ppm)

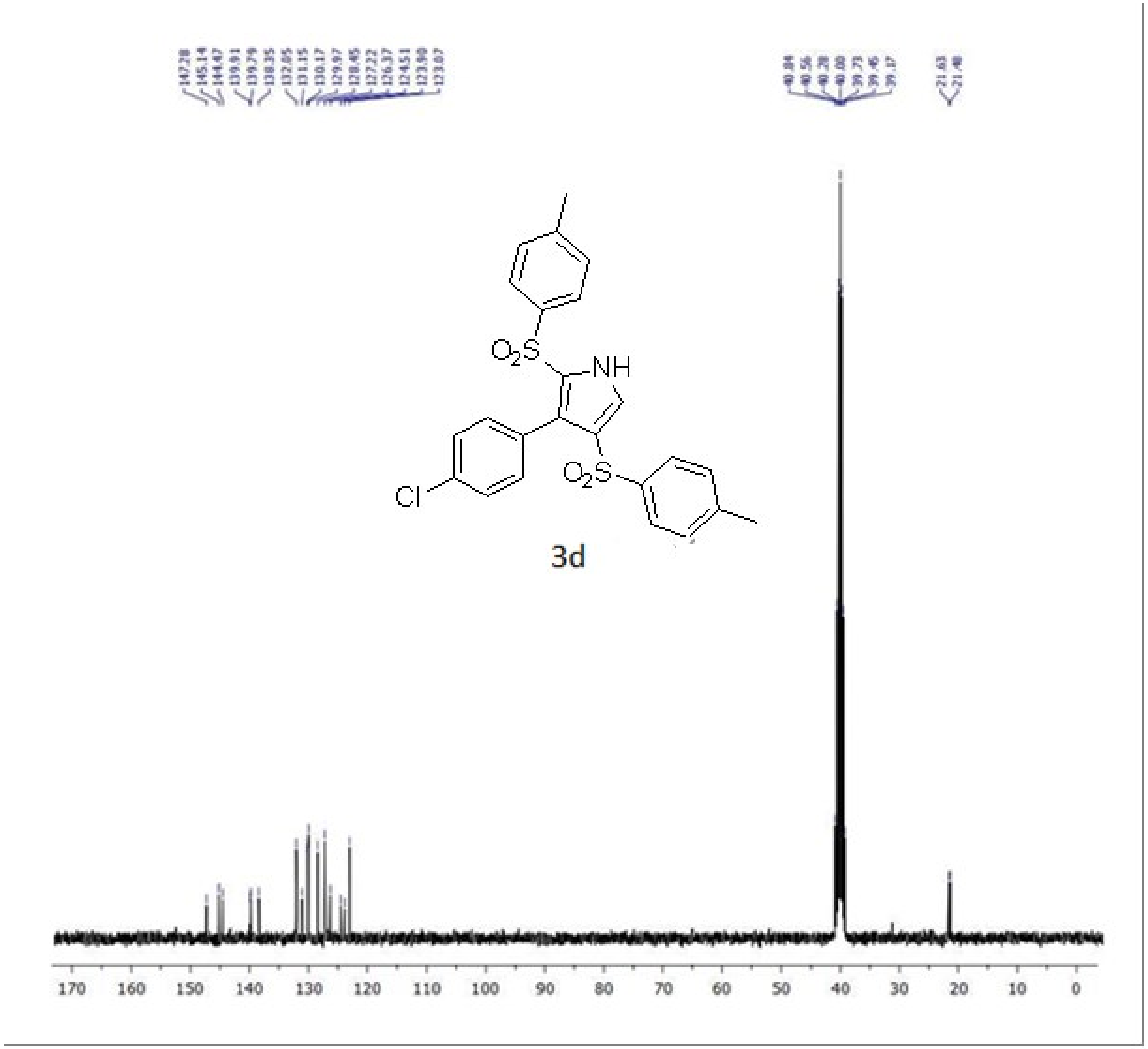

O2S
NH
Cl
O2S
3d
170 160 150 140 130 120 110 100 90 80 70 60 50 40 30 20 10 0

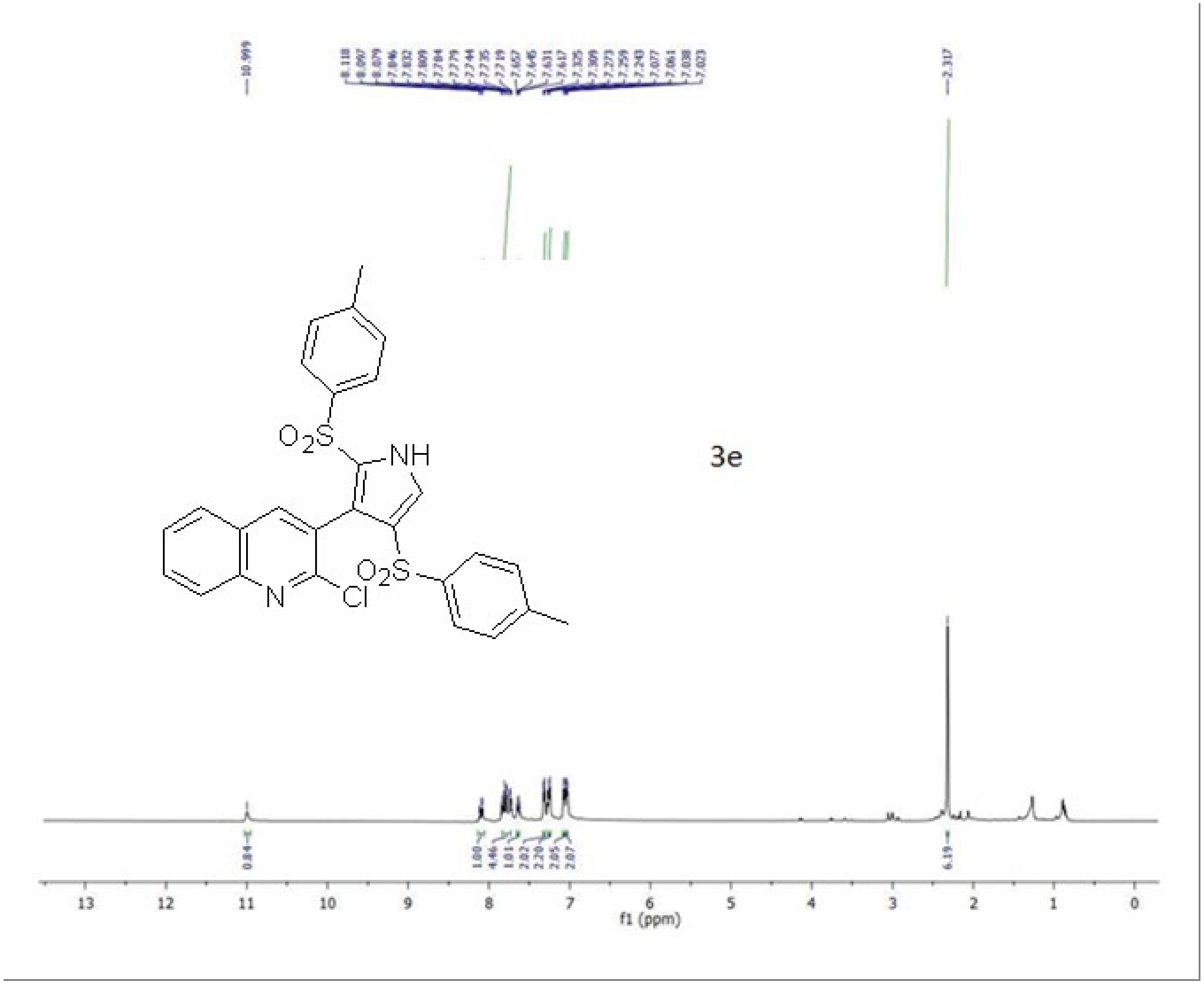

3e
f1 (ppm)

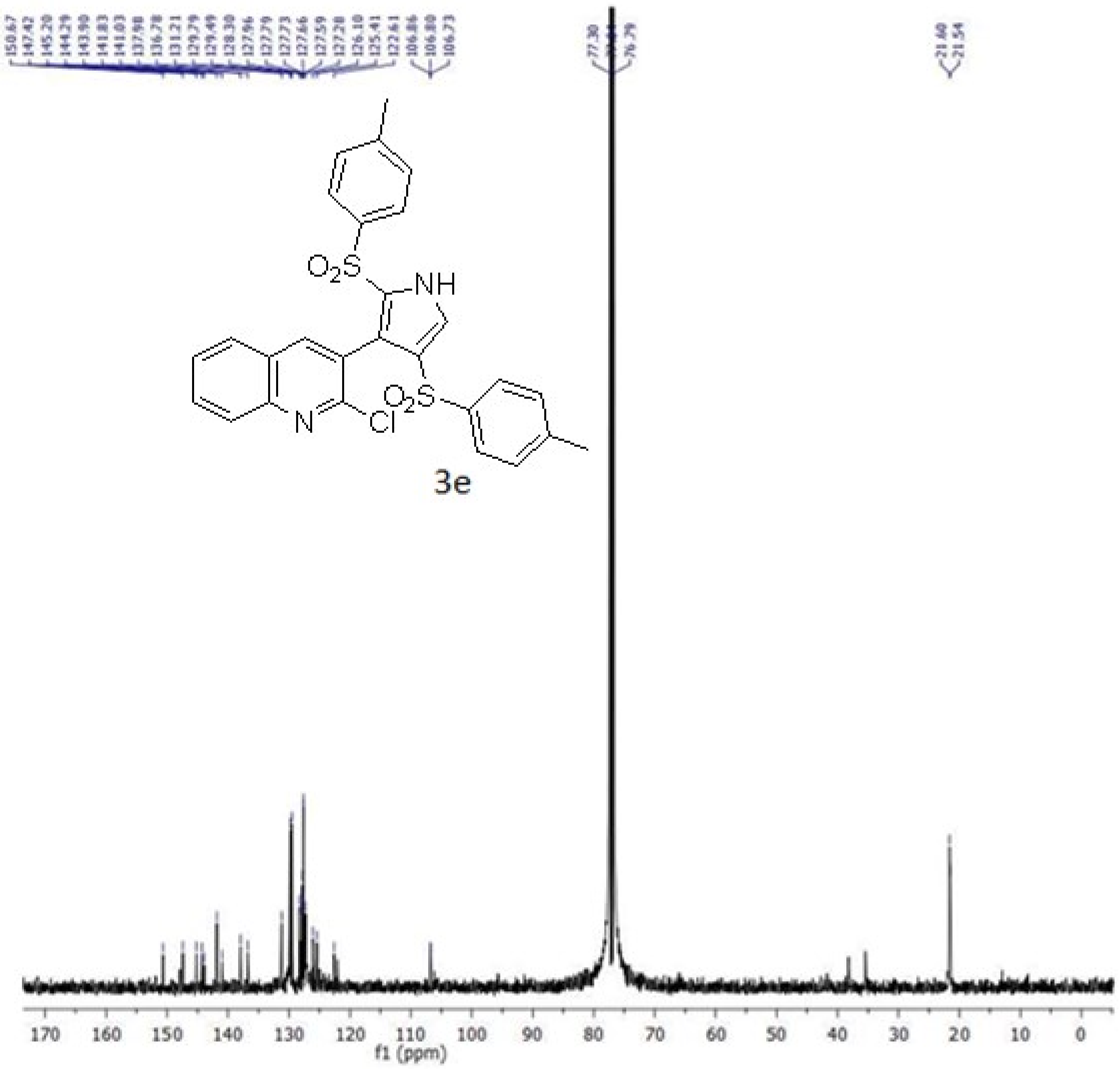
O2S
NH
N
Cl
O2S
3e
170 160 150 140 130 120 110 100 90 80 70 60 50 40 30 20 10 0
f1 (ppm)

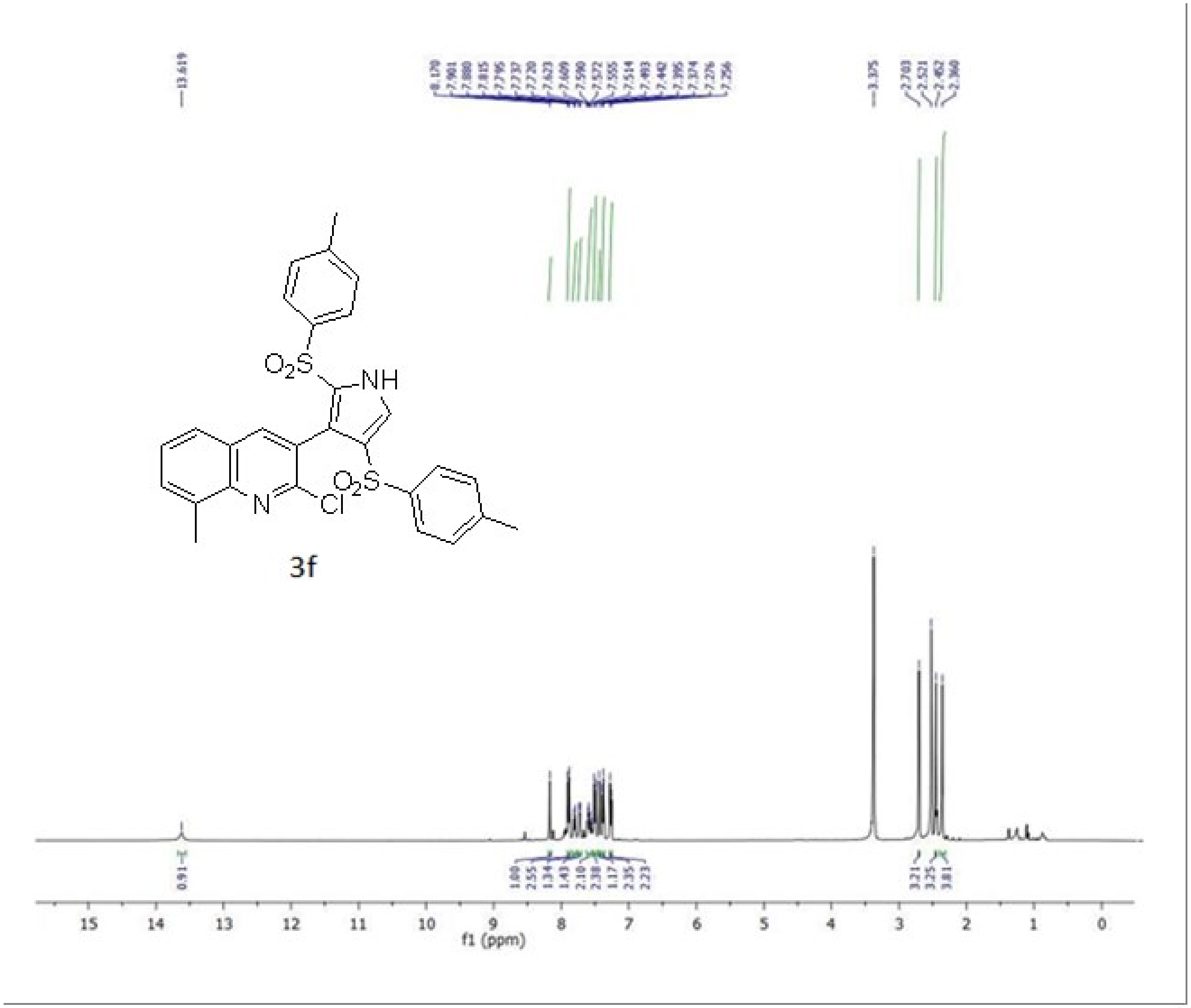
O2S
NH
N
Cl
3f
15 14 13 12 11 10 9 8 7 6 5 4 3 2 1 0
f1 (ppm)

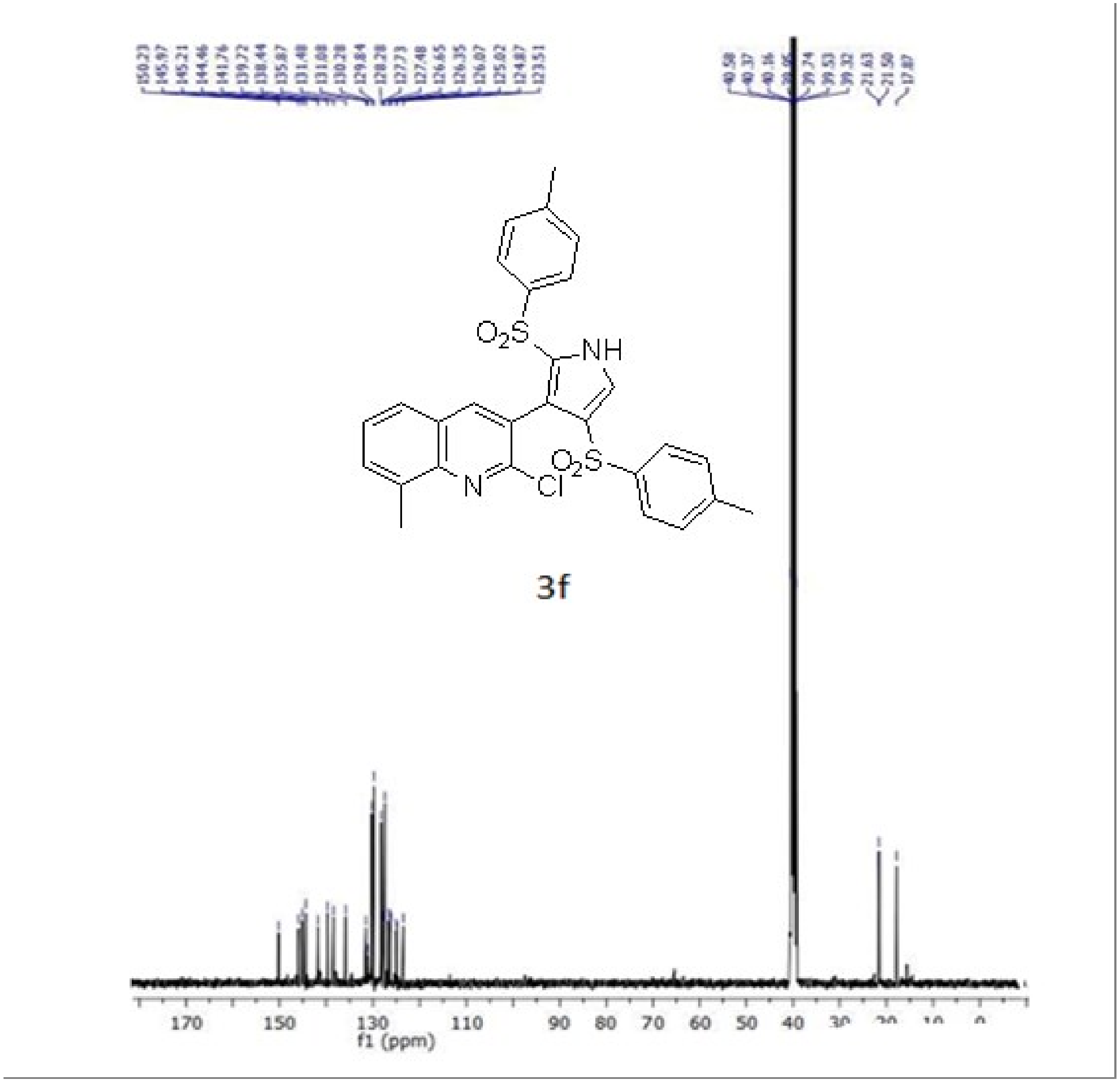

O2S
NH
O2S
N
Cl
3f
170
150
130
110
90
80
70
60
50
40
f1 (ppm)

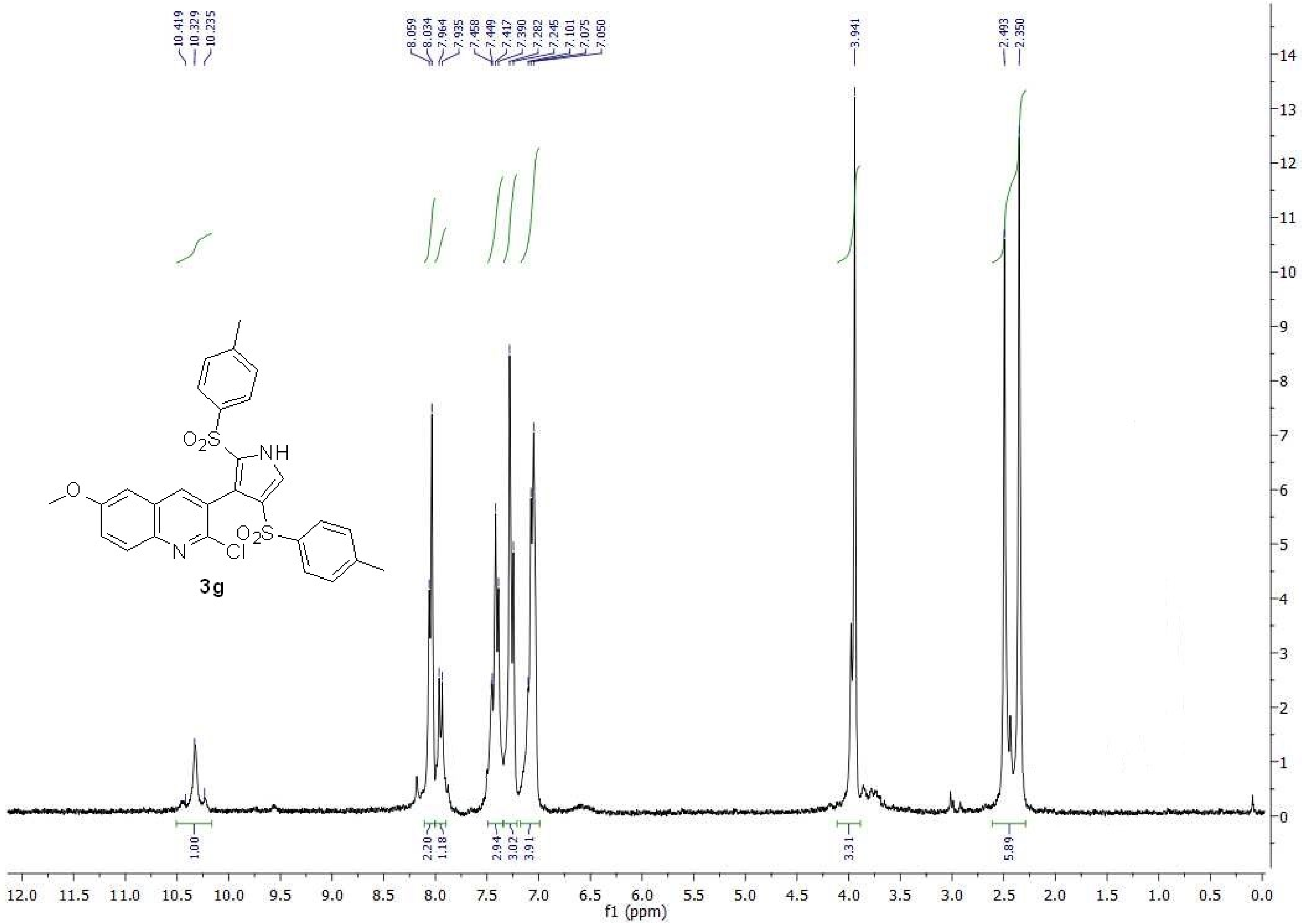
10.419
10.329
10.235
8.059
8.034
7.964
7.935
7.458
7.449
7.417
7.390
7.282
7.245
7.101
7.075
7.050
3.941
2.493
2.350
O2S
NH
O
N
Cl
O2S
3g
1.00
2.20
1.18
2.94
3.02
3.91
3.31
5.89
f1 (ppm)

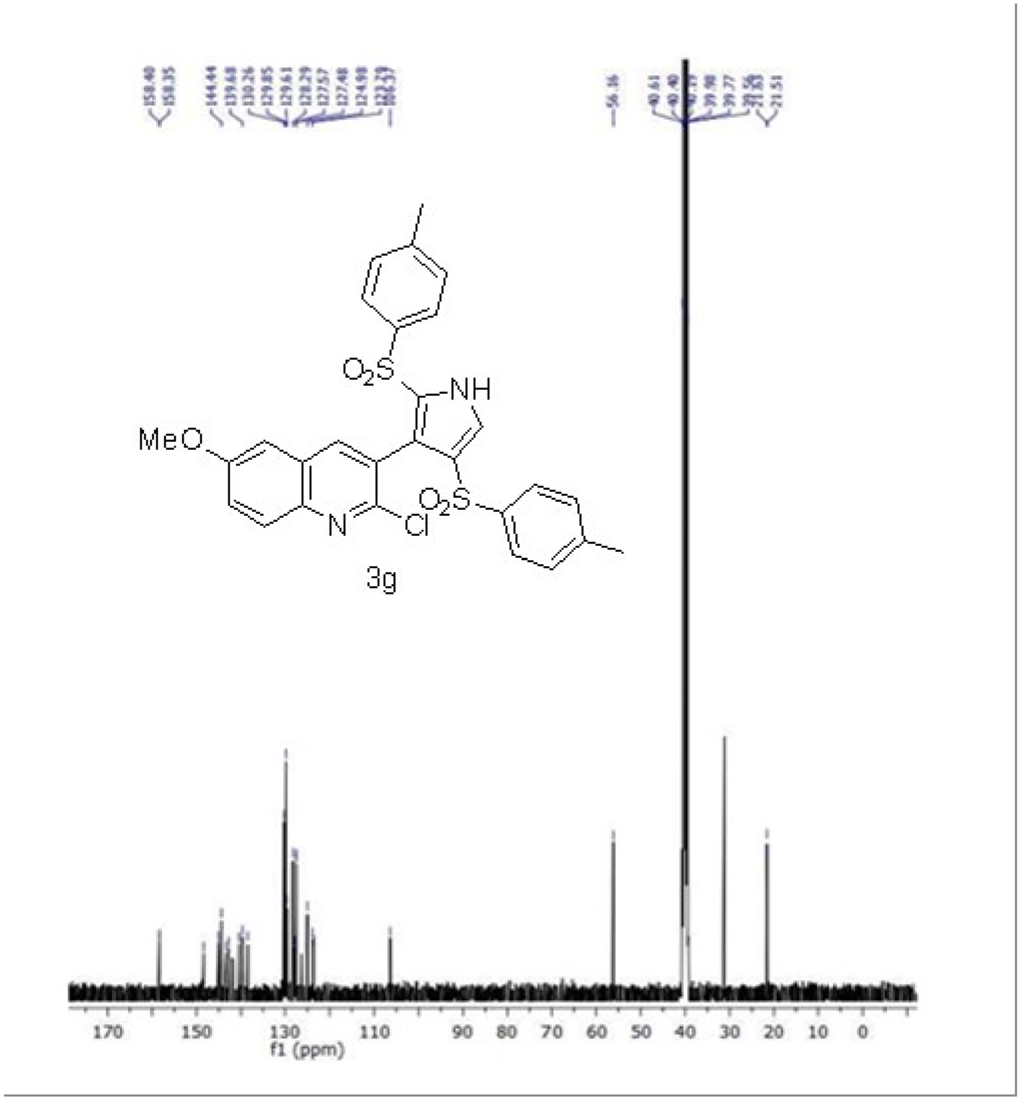
MeO
O_2S
NH
N
Cl
O_2S
3g
170 150 130 110 90 80 70 60 50 40 30 20 10 0
f1 (ppm)

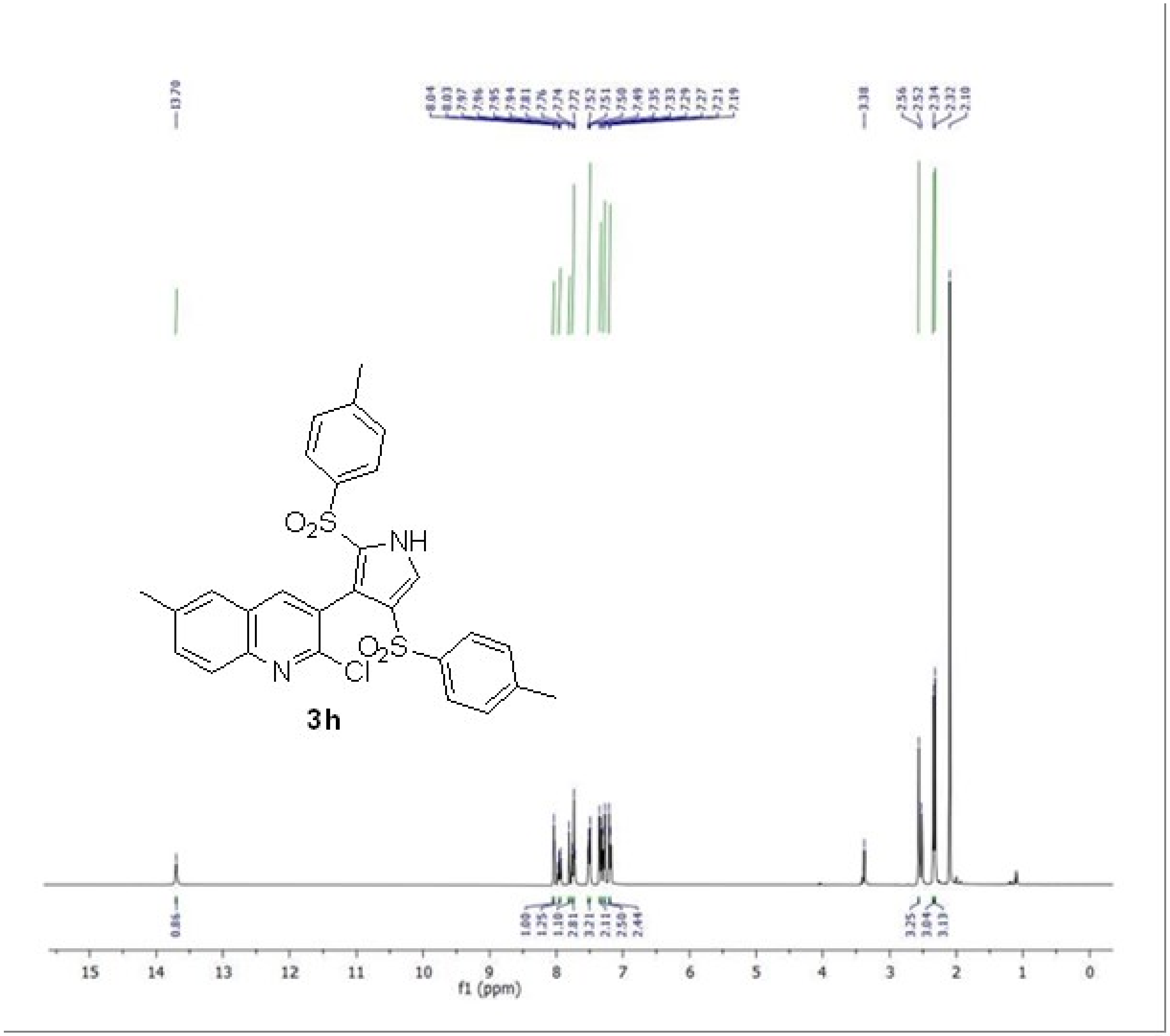

13.70
8.04
8.03
7.97
7.96
7.95
7.94
7.81
7.76
7.74
7.72
7.52
7.51
7.50
7.49
7.35
7.33
7.29
7.27
7.21
7.19
3.38
2.56
2.52
2.34
2.32
2.10
O2S
NH
N
Cl
O2S
3h
0.86
1.00
1.25
1.10
2.81
3.21
2.11
2.50
2.44
3.25
3.04
3.13
15
14
13
12
11
10
9
8
7
6
5
4
3
2
1
0
f1 (ppm)

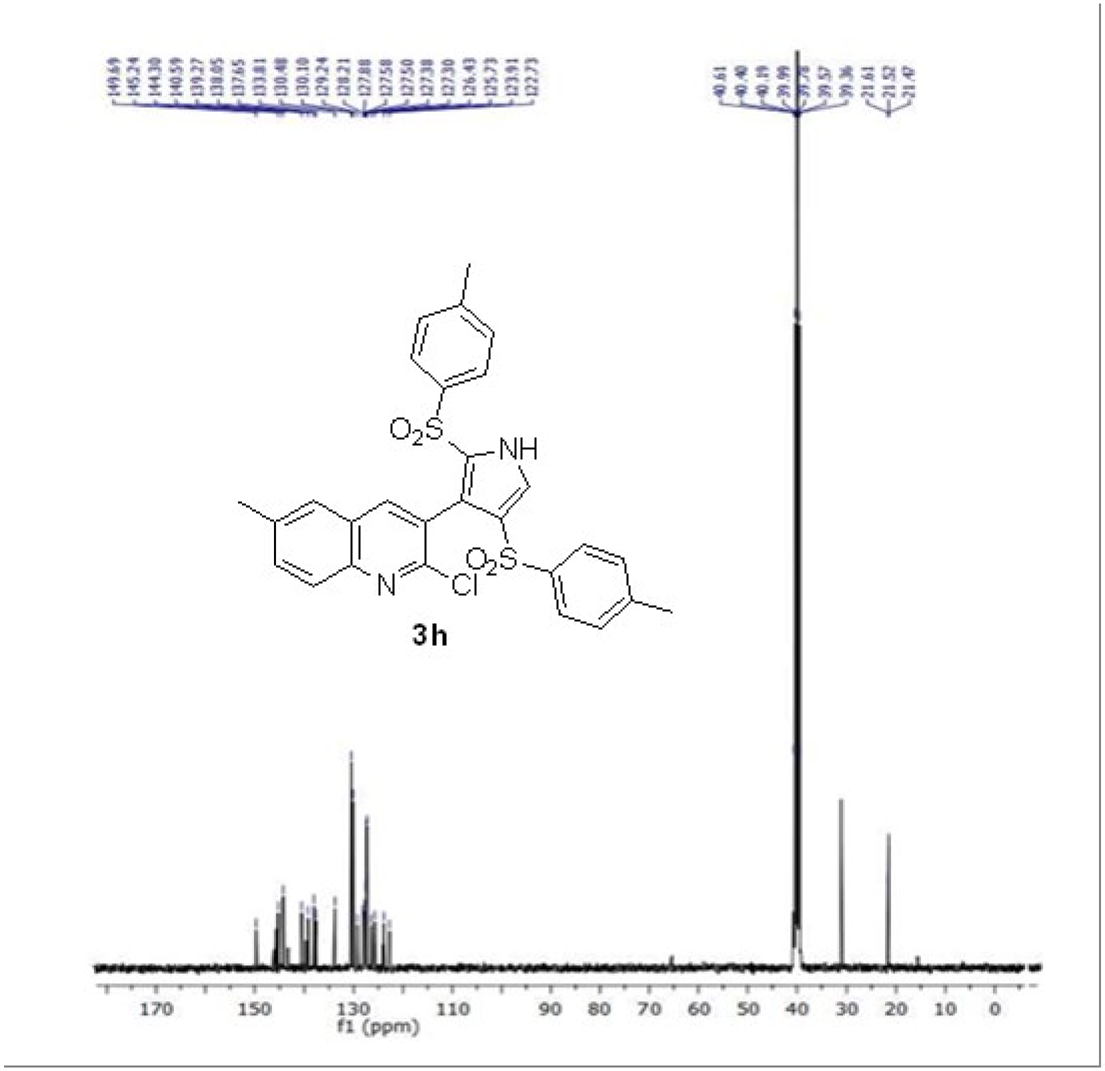

149.69
145.24
144.30
140.59
139.27
138.65
137.65
133.81
130.48
130.10
129.24
128.21
127.88
127.58
127.50
127.38
127.30
126.43
125.73
123.91
122.73
40.61
40.40
40.19
39.99
39.78
39.57
39.36
21.61
21.52
21.47
O2S
NH
N
Cl
3h
170
150
130
110
90
80
70
60
50
40
30
20
10
0
f1 (ppm)

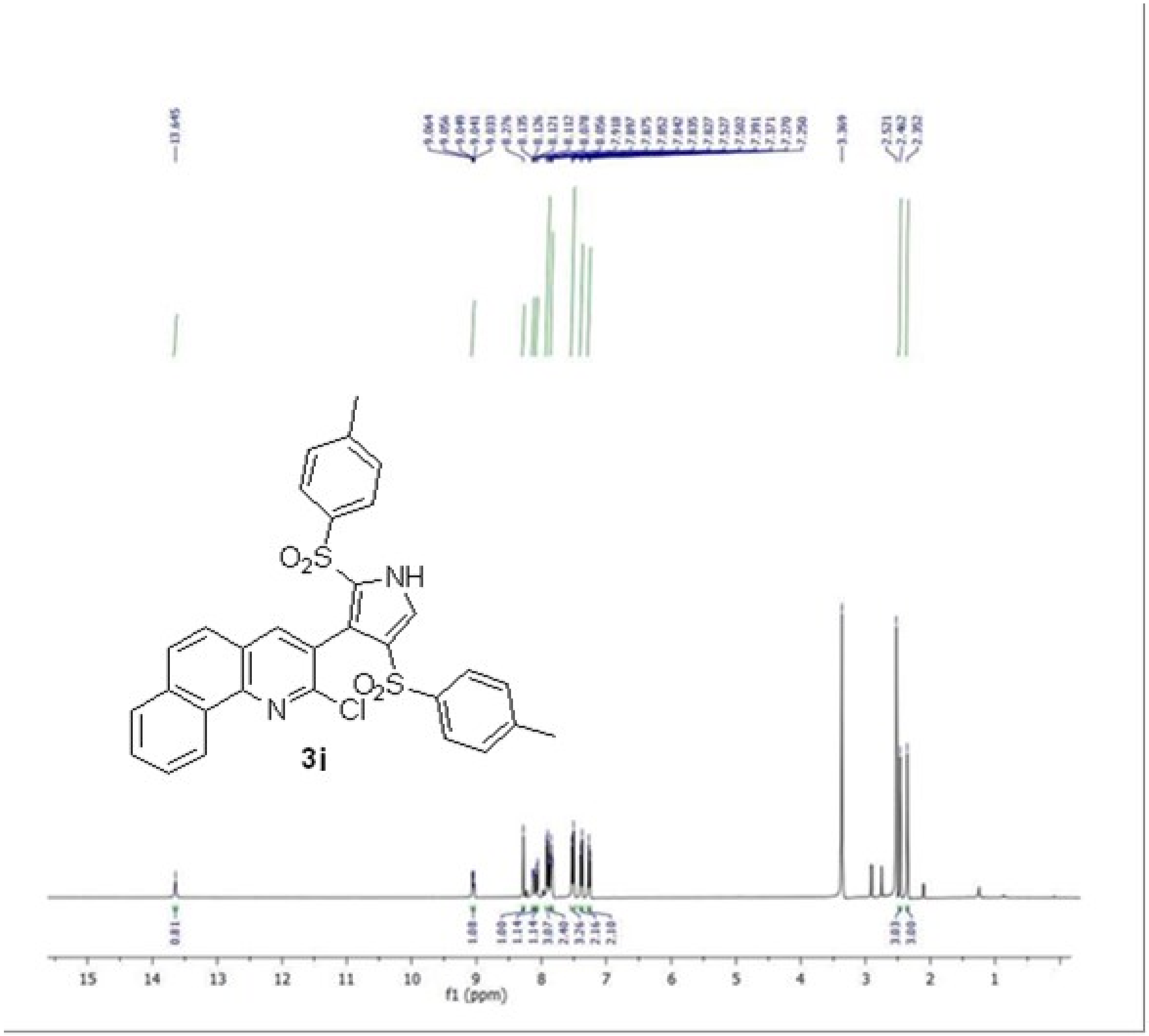
3i
f1 (ppm)

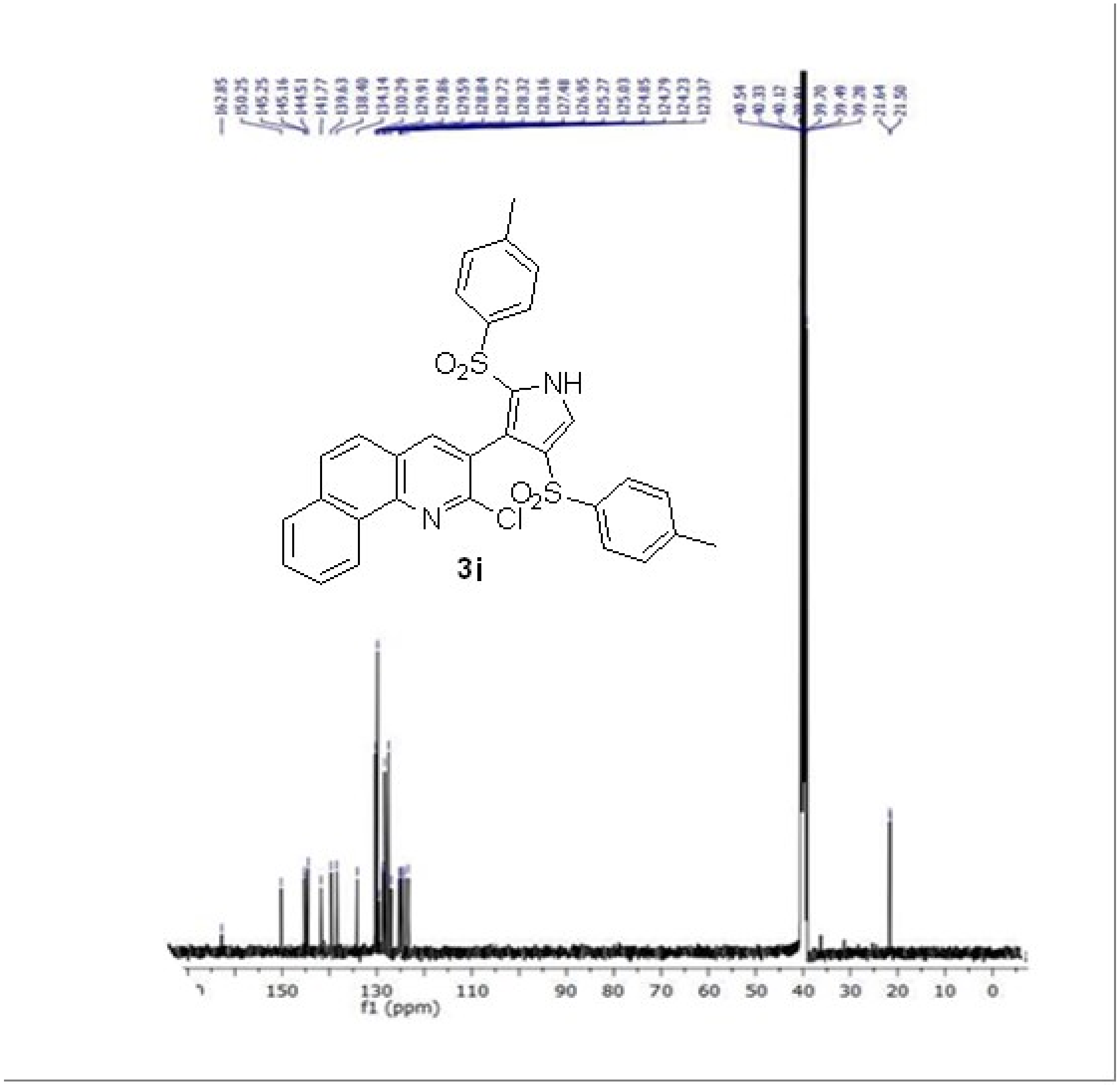

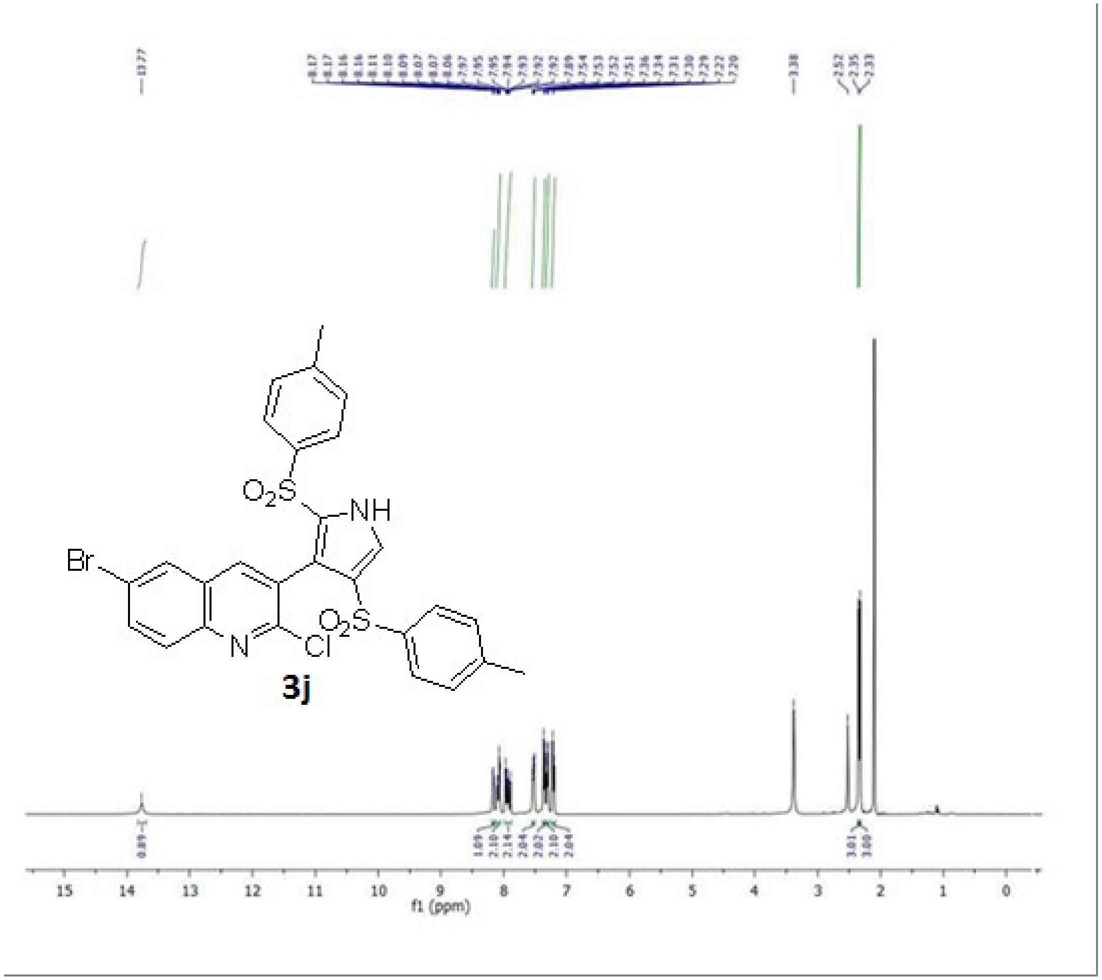

Br
N
Cl
NH
O_2S
O_2S
3j
f1 (ppm)

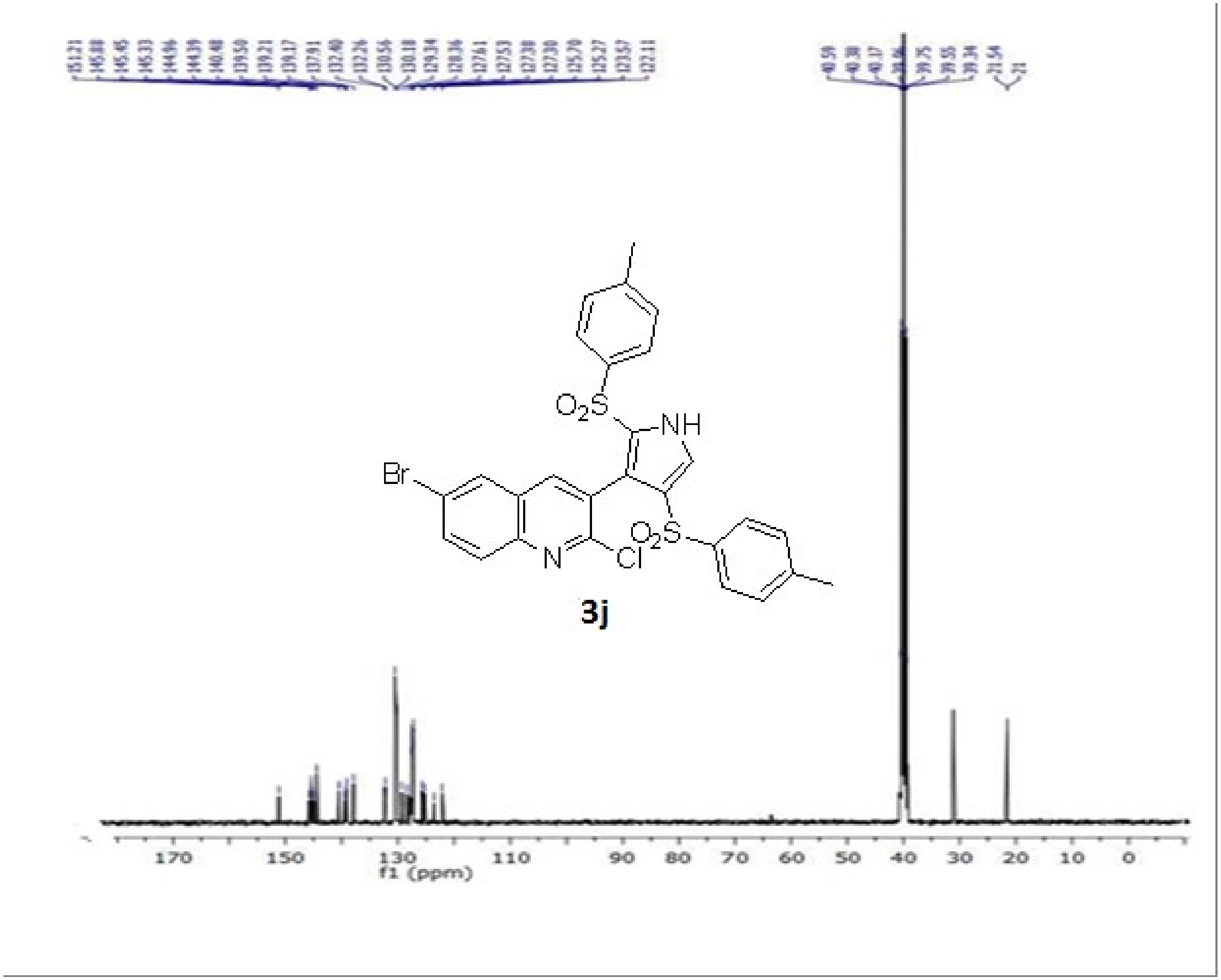

Br
N
Cl
NH
O_2S
O_2S
3j
170
150
130
110
90
80
70
60
50
40
30
20
10
0
f1 (ppm)

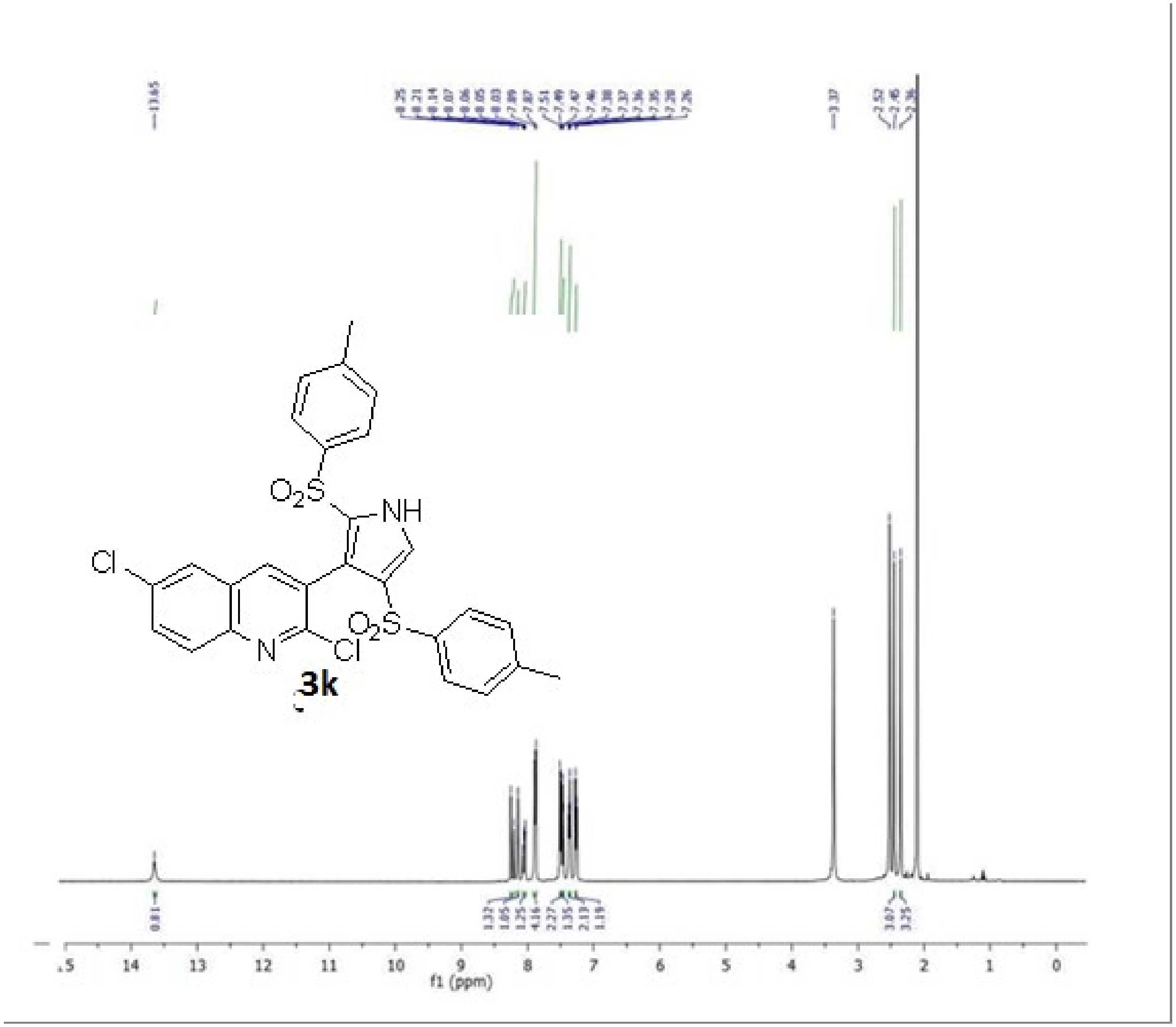

O2S
NH
Cl
N
Cl
O2S
3k
f1 (ppm)

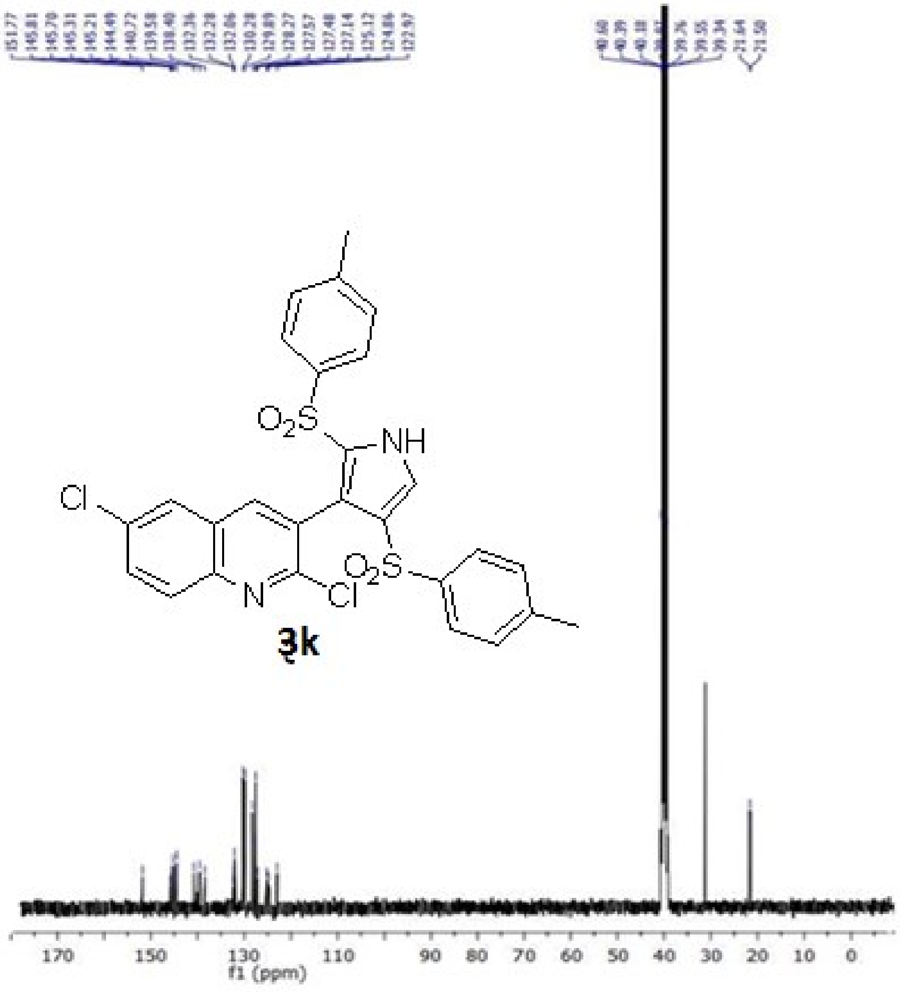
O2S
NH
Cl
N
Cl
O2S
3k
170 150 130 110 90 80 70 60 50 40 30 20 10 0
f1 (ppm)

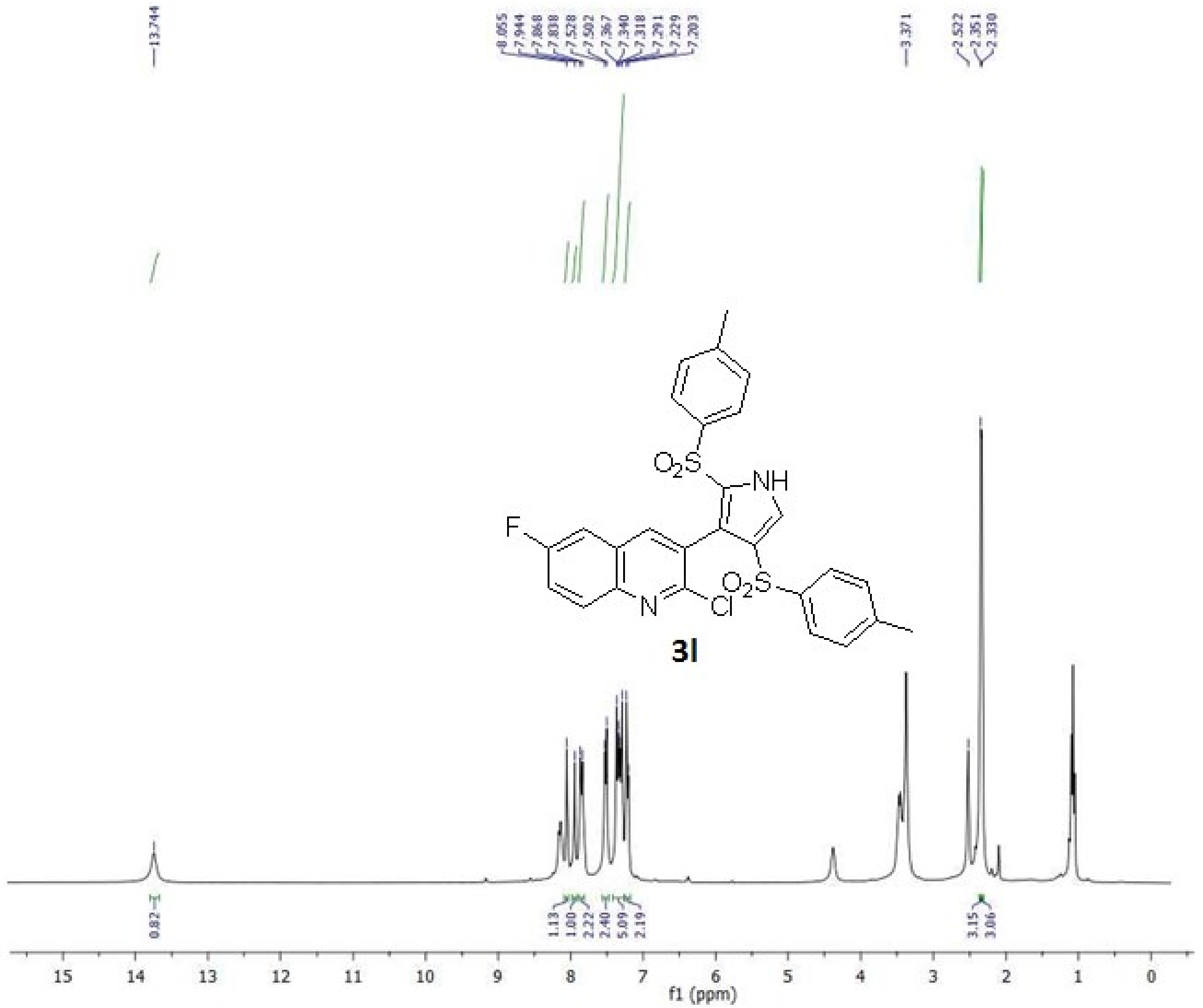
13.744
8.055
7.944
7.868
7.838
7.528
7.502
7.367
7.340
7.318
7.291
7.229
7.203
3.371
2.522
2.351
2.330
0.82
1.13
1.00
2.22
2.40
5.09
2.19
3.15
3.06
f1 (ppm)
3l

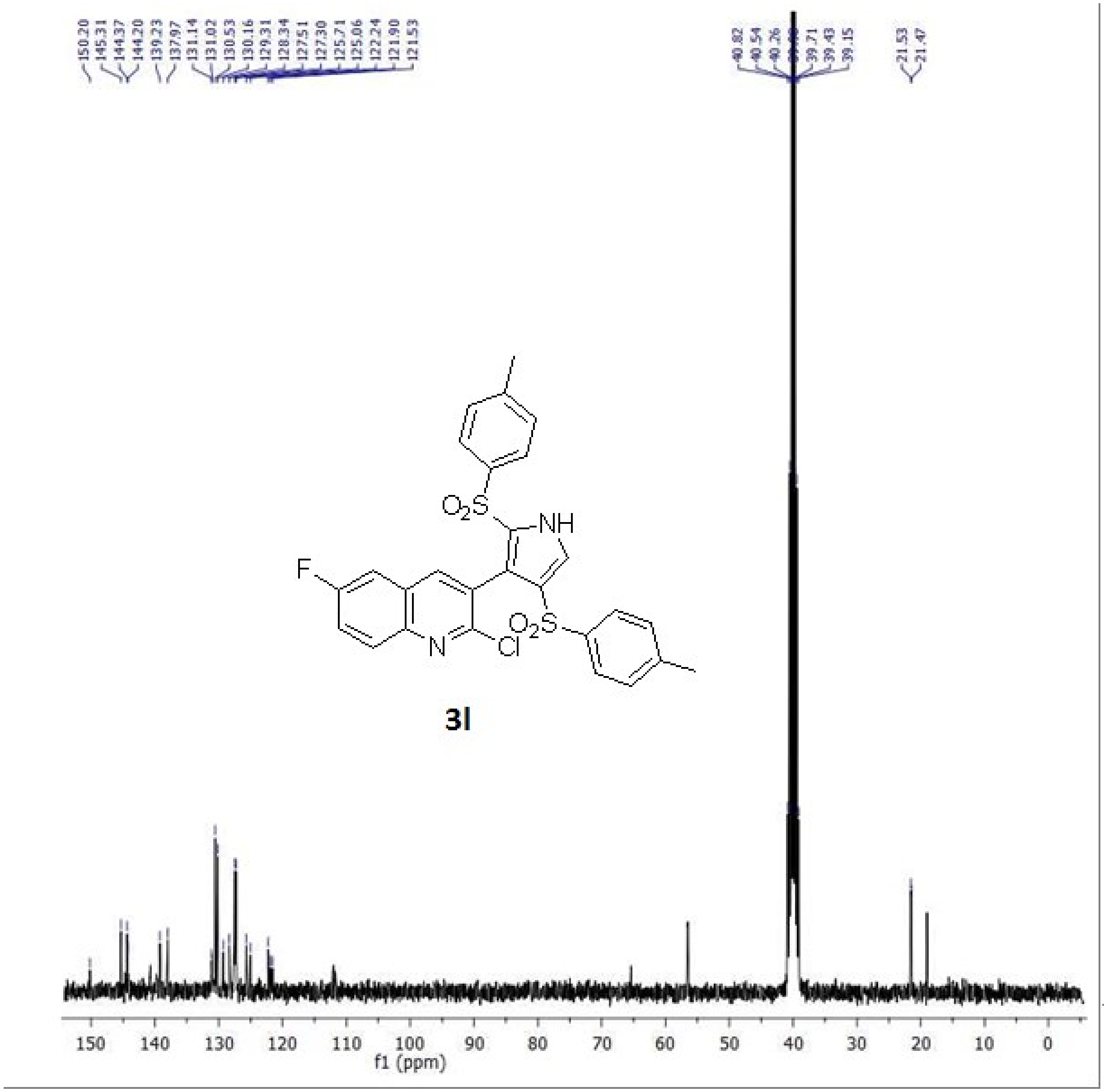
150.20
145.31
144.37
144.20
139.23
137.97
131.14
131.02
130.53
130.16
129.31
128.34
127.51
127.30
125.71
125.06
122.24
121.90
121.53
40.82
40.54
40.26
39.71
39.43
39.15
21.53
21.47
F
O_2S
NH
N
Cl
O_2S
3l
150
140
130
120
110
100
90
80
70
60
50
40
30
20
10
0
f1 (ppm)

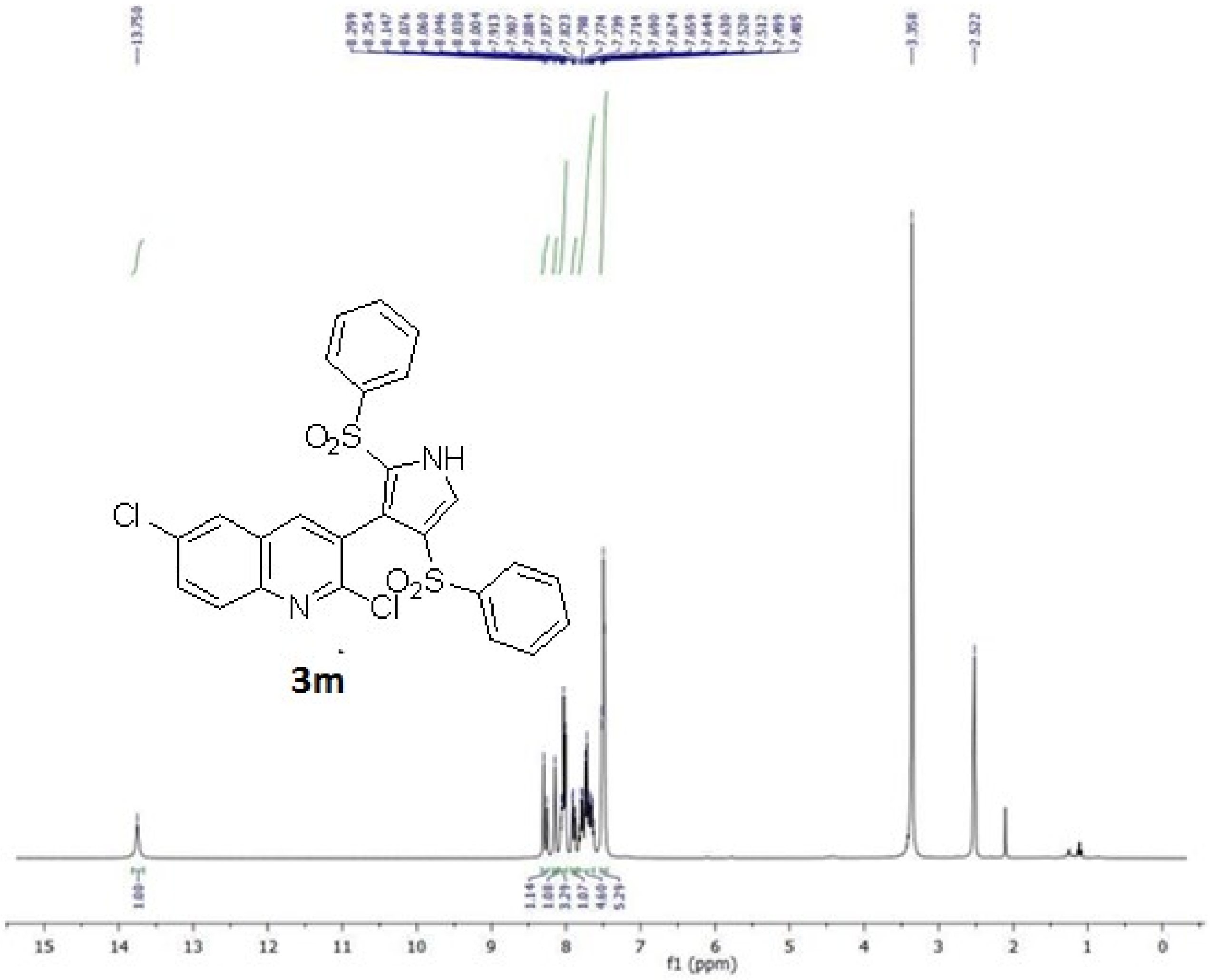

13.750
8.299
8.254
8.147
8.076
8.060
8.046
8.030
8.004
7.913
7.907
7.884
7.877
7.823
7.798
7.774
7.739
7.714
7.690
7.674
7.659
7.644
7.639
7.520
7.512
7.493
7.485
3.358
2.522
Cl
O2S
NH
N
Cl
O2S
3m
15
14
13
12
11
10
9
8
7
6
5
4
3
2
1
0
f1 (ppm)

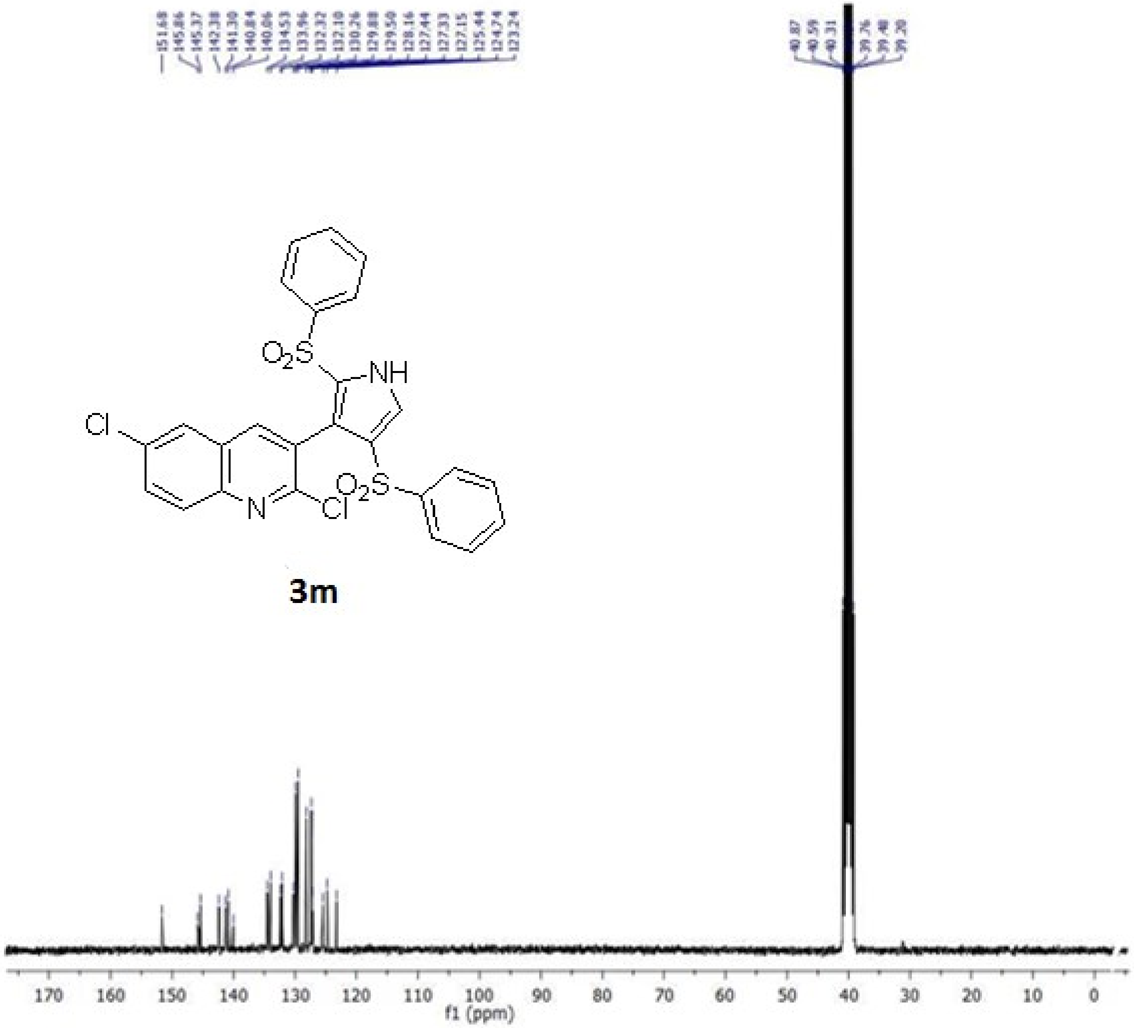
3m
Cl
N
Cl
NH
O2S
O2S
170 160 150 140 130 120 110 100 90 80 70 60 50 40 30 20 10 0
f1 (ppm)

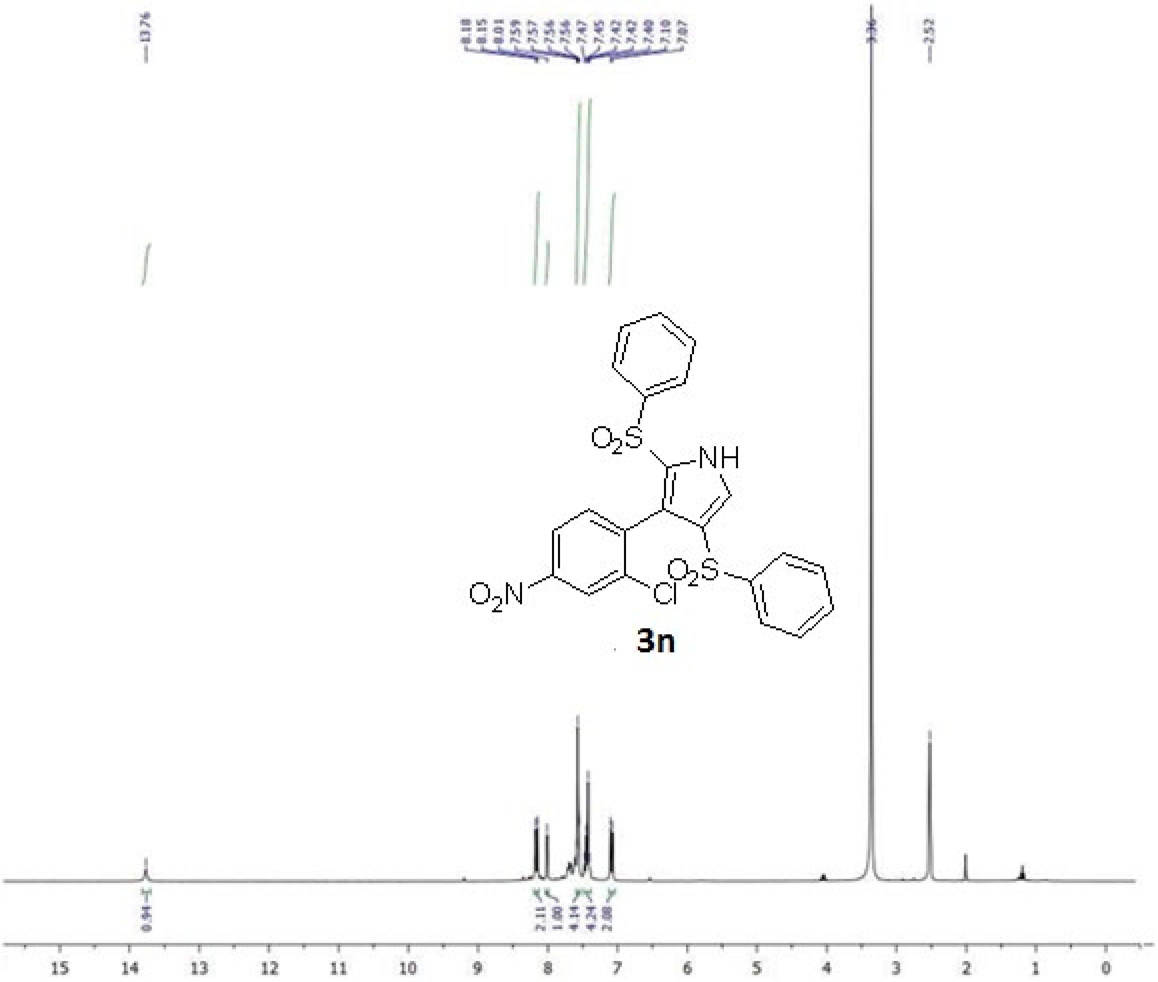

3n

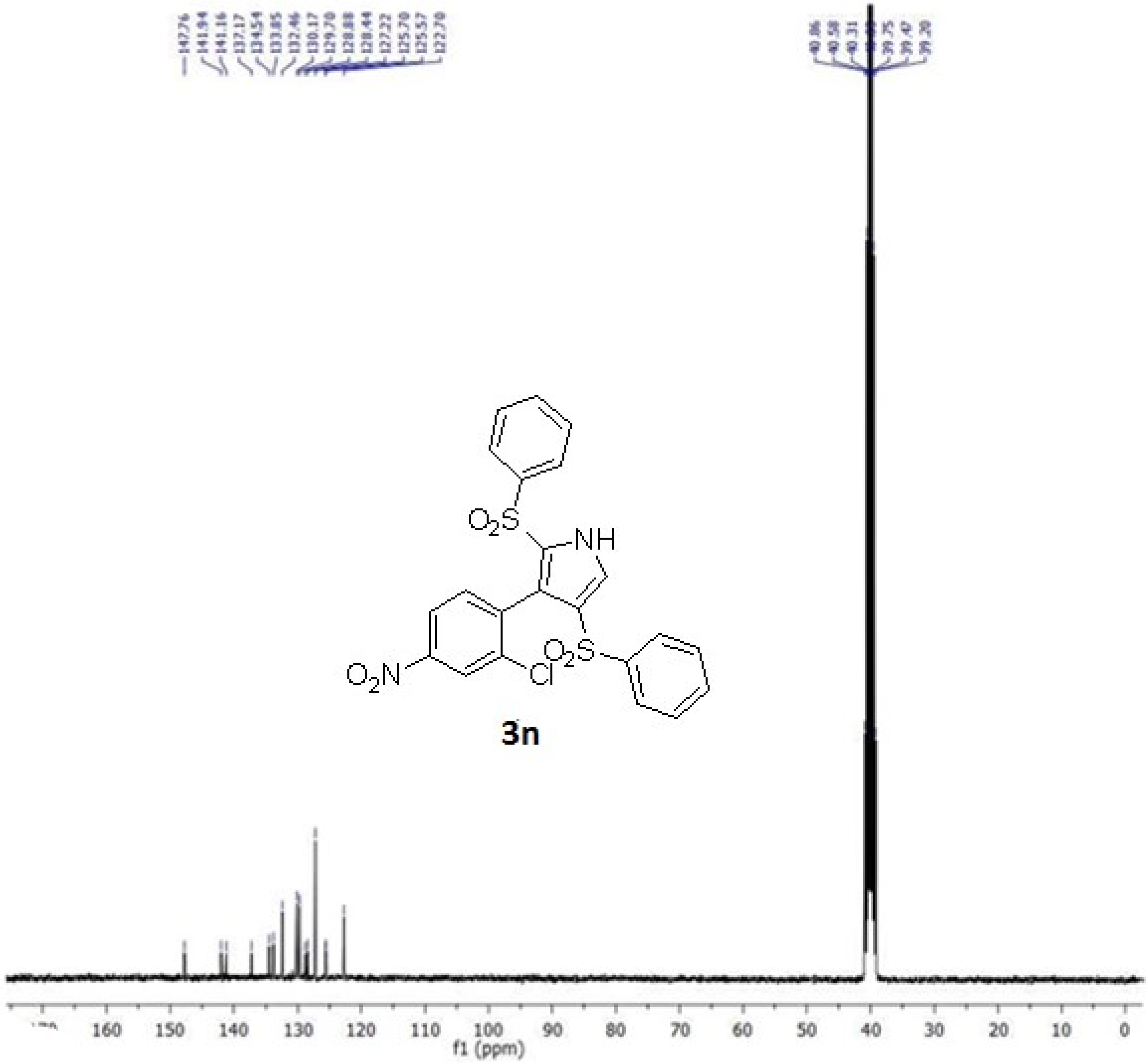
147.76
141.94
141.16
137.17
134.54
133.85
132.46
130.17
129.70
128.88
128.44
127.22
125.70
125.57
122.70
40.86
40.58
40.31
39.75
39.47
39.20
O2S
NH
O2N
Cl
O2S
160 150 140 130 120 110 100 90 80 70 60 50 40 30 20 10 0
f1 (ppm)

3n
